# Resolution-enhanced OCT and expanded framework of information capacity and resolution in coherent imaging

**DOI:** 10.1038/s41598-021-99889-3

**Published:** 2021-10-15

**Authors:** Nichaluk Leartprapun, Steven G. Adie

**Affiliations:** Nancy E. and Peter C. Meinig School of Biomedical Engineering, Cornell University, Ithaca, NY 14853 USA

**Keywords:** Imaging and sensing, Microscopy

## Abstract

Spatial resolution in conventional optical microscopy has traditionally been treated as a fixed parameter of the optical system. Here, we present an approach to enhance transverse resolution in beam-scanned optical coherence tomography (OCT) beyond its aberration-free resolution limit, without any modification to the optical system. Based on the theorem of invariance of information capacity, resolution-enhanced (RE)-OCT navigates the exchange of information between resolution and signal-to-noise ratio (SNR) by exploiting efficient noise suppression via coherent averaging and a simple computational bandwidth expansion procedure. We demonstrate a resolution enhancement of 1.5 × relative to the aberration-free limit while maintaining comparable SNR in silicone phantom. We show that RE-OCT can significantly enhance the visualization of fine microstructural features in collagen gel and ex vivo mouse brain. Beyond RE-OCT, our analysis in the spatial-frequency domain leads to an expanded framework of information capacity and resolution in coherent imaging that contributes new implications to the theory of coherent imaging. RE-OCT can be readily implemented on most OCT systems worldwide, immediately unlocking information that is beyond their current imaging capabilities, and so has the potential for widespread impact in the numerous areas in which OCT is utilized, including the basic sciences and translational medicine.

## Introduction

The enhancement of resolution^1–5^ has been an important and on-going pursuit in all fields of imaging, including coherent^6–32^ and incoherent^33–44^ optical microscopy. One approach for resolution enhancement is spatial-frequency bandwidth expansion [i.e., increasing the numerical-aperture (NA)] via aperture synthesis. This family of techniques utilizes multiple measurements of the sample that provide access to different spatial frequencies beyond the bandwidth support of a single measurement. A well-known technique in incoherent microscopy is structured illumination microscopy (SIM), which uses different illumination patterns to shift the spatial frequency coverage of the optical system^37–39^. Virtually structured detection (VSD) adopts a similar principle but (virtually) applies a digital spatial modulation in the detection path^40^. In coherent microscopy, the bandwidth support of the optical system can be shifted via use of multiple illumination angles, as has been implemented with off-axis holography^6–8^ and Fourier ptychography^9–11^ (which performs incoherent imaging at different illumination angles, combined with phase retrieval methods to reconstruct the complex optical field).

In optical coherence tomography (OCT), interferometric synthetic aperture microscopy (ISAM) utilizes synthetic aperture methods to overcome the trade-off between resolution and depth-of-field, in order to reconstruct depth-invariant focal-plane resolution from a single volumetric measurement with the optical focus at a fixed depth^12,13^. Combining full-field OCT with holography, holoscopy can similarly achieve focal-plane resolution across all depths^14^. The VSD approach has also been applied to OCT^15^. More recently, optical coherence refraction tomography (OCRT) utilizes sample rotation combined with an (incoherent) Fourier synthesis technique reminiscent of X-ray computed tomography to effectively ‘replace’ the lateral resolution of a low-NA imaging beam by the superior axial resolution of OCT^16^. Similarly, sample shifting (sub-resolution translation) has been combined with multi-frame super-resolution image processing to reconstruct an OCT tomogram with improved resolution^17^. Meanwhile, deconvolution approaches based on iterative numerical optimization (of OCT signal magnitude or intensity images) have also been implemented to enhance resolution in OCT^18–20^.

Another family of techniques aims to correct aberrations in order to restore ideal focal-plane resolution. Hardware-based adaptive optics (HAO) has been implemented in coherent^21–24^ and incoherent^33–36^ microscopy. In coherent imaging, access to the complex optical field can enable computational aberration correction post-data-acquisition. Computational adaptive optics (CAO) modifies the pupil phase of complex OCT tomograms to correct both defocus and optical aberrations, in order to restore aberration-free focal-plane resolution^25–32^. Indeed, reaching the ideal aberration-free resolution supported by the optical system (i.e., without entering the super-resolution regime), especially in complex media such as biological samples, is the aim of many adaptive optics or computational aberration correction methods in optical microscopy.

Spatial resolution (or equivalently, spatial-frequency bandwidth) in optical microscopy has traditionally been treated as a *fixed* parameter for a given optical system. However, the *theorem of invariance of information capacity* suggests that resolution of an optical system is a *tuneable* parameter that can be flexibly modified *without* altering the optical system^3–5^. Cox and Sheppard described the information capacity of an optical system as the product of its space-bandwidth products (SBP) over all spatial dimensions, time-bandwidth product (TBP), and its signal-to-noise ratio (SNR) on the logarithmic scale^4^. The theorem of invariance of information capacity states that it is not the spatial-frequency bandwidth (and therefore resolution), but the information capacity of an optical system that is invariant^3–5^. It follows that spatial resolution can, in theory, be enhanced beyond the aberration-free limit of a given optical system through an exchange of information between spatial-frequency bandwidth and SNR, while keeping the information capacity constant.

The framework of informational capacity and its invariance underscores several unique advantages of coherent over incoherent imaging. Given the same number of pixels, a coherent image inherently supports twice the information capacity of an equivalent incoherent image because each pixel is described by both magnitude and phase of the complex optical field, as opposed to a single intensity value^4,5,45^. Using quantum Fisher information formalism, others have also shown that the resolution limit in traditional intensity-based imaging techniques can be overcome by making use of phase information^46–48^. Bilenca et al. derived the scattering limit to the information capacity of depth-resolved coherent imaging (using OCT as a case study) through turbid media as a function of SNR^49^. Furthermore, coherent averaging of complex tomograms provides a more efficient method for noise suppression than incoherent (magnitude-only) averaging in OCT, due to the decorrelation of random phase noise^50–52^. Coherent averaging has also been used for multiple scattering suppression^53–55^. The efficient noise suppression via coherent averaging presents an opportunity to expand the information capacity of a coherent imaging system via the enhancement of SNR.

Resolution-enhanced (RE)-OCT is a spatial-frequency bandwidth expansion approach that computationally enhances transverse spatial frequencies beyond the traditional bandwidth support (i.e., beyond the aberration-free resolution limit) of a beam-scanned OCT system. Unlike existing aperture synthesis techniques^6–11,16,37–39^, it does not rely on diversity in the illumination schemes to access traditionally unseen spatial frequencies, and so can readily be implemented on existing OCT systems. RE-OCT is based upon the premise of information exchange between spatial-frequency bandwidth and SNR, as governed by the theorem of invariance of information capacity^3–5^. It navigates this exchange of information by exploiting efficient noise suppression via coherent averaging and a simple computational bandwidth expansion procedure. RE-OCT harnesses the benefit of coherent averaging to (for the first time) enhance resolution in OCT.

In this paper we first discuss the underlying principle of RE-OCT based on the information capacity framework. Then, we demonstrate noise suppression via coherent averaging and analyse its impact on the OCT signal in not only the space, but also the spatial-frequency domain. Our analysis of the impact of coherent averaging in the transverse spatial-frequency domain provides a new perspective compared to prior work in OCT (which has only investigated the impact of coherent and incoherent averaging in the space domain^50–52^). Next, we demonstrate resolution enhancement by RE-OCT in both resolution phantom and biological samples (collagen gel and ex vivo mouse brain). We then analyse the factors that limit resolution enhancement in RE-OCT by comparing experimental results to simulations. Lastly, we leverage insights from our analysis in the spatial-frequency domain to present an expanded framework of information capacity and resolution in coherent imaging. RE-OCT has the potential to have widespread impact since it can be readily implemented on most OCT systems without requiring any redesign of the optical system or specialized computationally-intensive algorithms.

## Results

### Underlying principle of resolution-enhanced OCT

**Figure 1:**
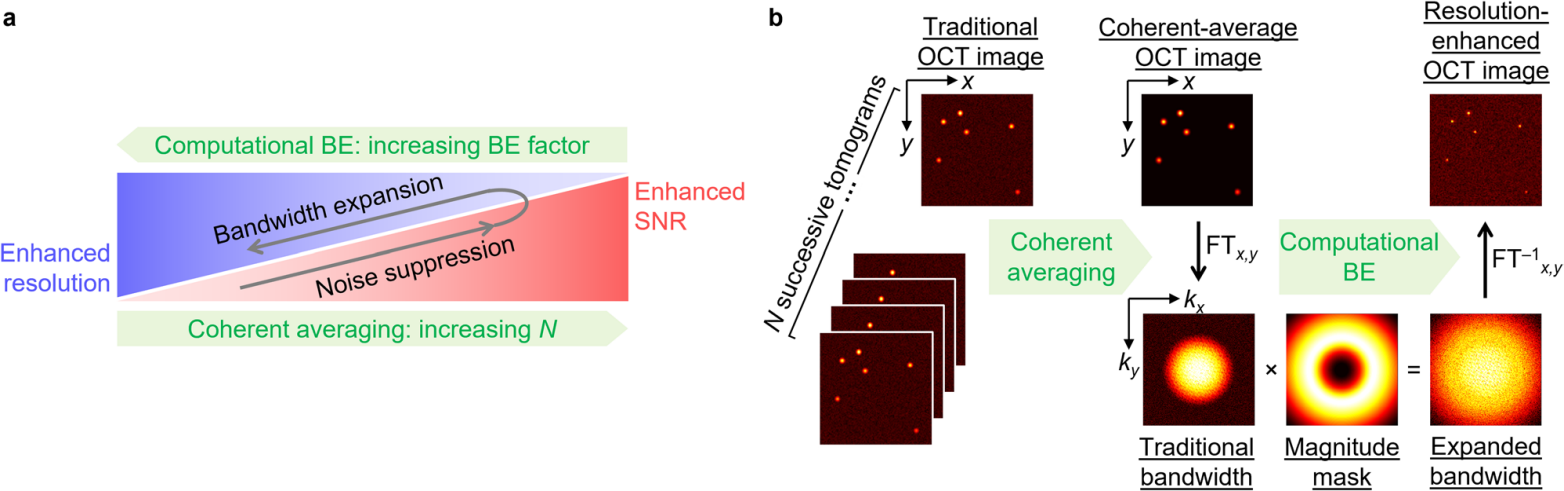
Underlying principle of RE-OCT. (**a**) Information exchange between resolution and SNR facilitated by noise suppression and spatial-frequency bandwidth expansion. RE-OCT utilizes coherent averaging to efficiently suppress noise and enhance SNR, then SNR is sacrificed in the computational BE procedure to enhance resolution. (**b**) Illustration of RE-OCT process to implement the principle in (**a**). Supplementary Section IV provides a full description of RE-OCT reconstruction procedure. *BE* bandwidth expansion, *FT* Fourier transform.

RE-OCT is a spatial-frequency bandwidth expansion approach based on the exchange of information between resolution and SNR that is supported by the underlying theorem of invariance of information capacity^3–5^. We conjecture that the transverse resolution of a tomogram acquired by a beam-scanned OCT system can be enhanced via computational bandwidth expansion (BE), where signal at higher spatial frequencies is raised via multiplication by a magnitude mask in the spatial-frequency domain (i.e., magnitude-based deconvolution). This is feasible due to the under-filling of the physical aperture of the objective lens (i.e., a physical bandwidth limit) that is implemented in the ubiquitous telecentric scanning scheme (Supplementary Fig. 2b) used by most OCT systems worldwide. However, deconvolution inherently amplifies noise and degrades the SNR of the image. To address this problem, we also conjecture that the SNR penalty associated with computational BE can be compensated by first suppressing the system noise—one efficient way in OCT is by coherently averaging multiple successively acquired complex tomograms^50–52^. The underlying principle of RE-OCT is illustrated in Fig. 1. RE-OCT utilizes coherent averaging for efficient noise suppression to ‘earn’ SNR, which can be used to ‘purchase’ resolution via computational BE.

To understand the underlying principle of RE-OCT in the context of the theorem of invariance of information capacity, we restate Cox and Sheppard’s expression for the information capacity of an optical system^4^ (Eq. 1).

1 $$C = \left( {2L_{x} B_{x} + 1} \right)\left( {2L_{y} B_{y} + 1} \right)\left( {2L_{z} B_{z} + 1} \right)\left( {2TB_{T} + 1} \right)\log_{2} \left( {1 + {s \mathord{\left/ {\vphantom {s n}} \right. \kern-\nulldelimiterspace} n}} \right),$$

where *L*, *T*, and *B* denote the spatial field-of-view (FOV), temporal duration, and bandwidth in the associated dimension, respectively. The first three terms represent the SBP along the three spatial dimensions while the fourth term represents the TBP. The last term represents the SNR (in bits), where *s* and *n* denote the average signal power and the additive noise power, respectively. Consider simply acquiring *N* successive tomograms of a ‘static’ object. Although *T* increases by a factor of *N*, the TBP remains constant because the object is known a priori to be *invariant* in time (i.e., infinitesimal *B*_*T*_)—no additional information is derived from the object despite the capacity to support more information. By coherently averaging the *N* successive tomograms, however, the originally redundant increase in *T* is now “encoded” in the finite SNR term via the reduction in *n*. Thus, compared to the individual tomograms, the new coherent-averaged system possesses a larger information capacity with enhanced SNR (Supplementary Eq. S4), which can be sacrificed to expand each of the transverse spatial-frequency bandwidths, *B*_*x*_ and *B*_*y*_. (For the full analysis of this process, see Supplementary Section I). One scenario is to expand the bandwidth equally along each transverse dimension by a factor equal to the square root of the gain in SNR in order to, in principle, enhance resolution in the transverse plane by the same factor, without suffering any SNR penalty relative to the traditional single-shot tomogram (Supplementary Fig. 1). However, RE-OCT is not limited to this scenario; the trade-off between resolution and SNR can be flexibly navigated by tuning the number of tomograms averaged and the BE factor applied (i.e., more SNR than the amount ‘earned’ from coherent averaging can be sacrificed to prioritize further resolution enhancement) (Fig. 1a).

### Coherent-average noise suppression in space and spatial-frequency domains

**Figure 2:**
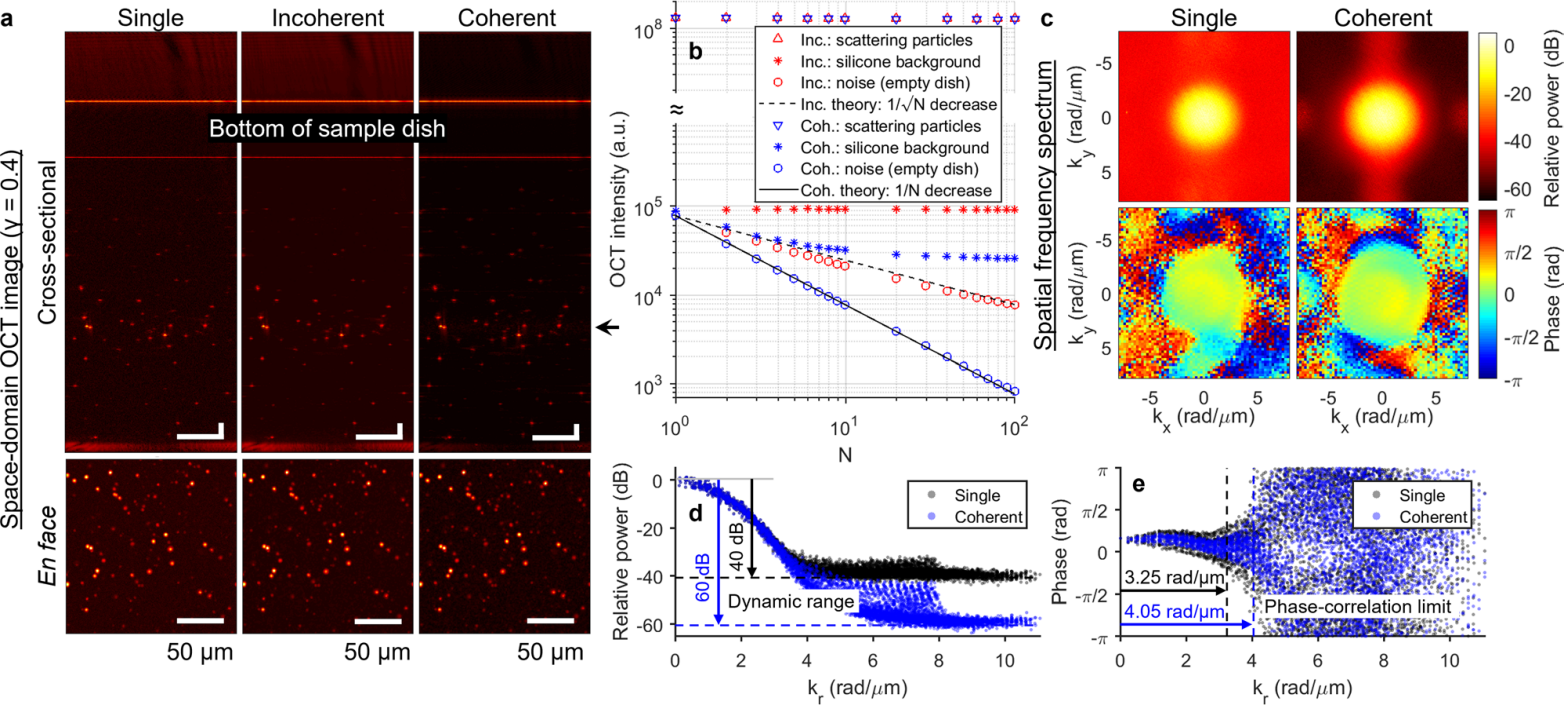
Coherent-average noise suppression with 100 acquisitions in silicone phantom. (**a**) Single-shot, incoherent- and coherent-average OCT images (same colormap range). En face images correspond to focal plane, indicated by arrow. Scale bar, 50 µm. (**b**) OCT intensity of scattering particles, silicone background, and noise as a function of *N* for incoherent (red) and coherent (blue) average. Noise intensity was obtained from the “noise image” of an empty sample dish (see “Methods”), at the same pixel depth as the focal plane of the phantom images. (**c**) Power and phase of single-shot and coherent-average images in transverse spatial-frequency domain (with spatial frequencies *k*_*x*_ and *k*_*y*_). Phase spectrum was obtained from the Fourier transform of a windowed region around a single particle. (**d**) Power spectrum in (**c**) plotted as a function of radial transverse spatial frequency *k*_*r*_. Dotted line indicates noise floor obtained from power spectrum of the noise image. (**e**) Phase spectrum in (**c**) plotted as a function of *k*_*r*_. Dotted line indicates *k*_*r*_ limit beyond which phase becomes decorrelated (local standard deviation > 0.2 rad, see “Methods”). Supplementary Section VII investigates relations between *N*, DR and phase-correlation limit. Calculations of OCT intensity, DR, and phase-correlation limit are described in “Methods”.

Noise suppression via coherent and incoherent average over *N* = 1 through 100 acquisitions was investigated in both space and spatial-frequency domain in a silicone phantom containing scattering particles (Fig. 2). Factors that contributed to the measured noise include widely known sources of noise in OCT (i.e., shot noise, thermal noise, laser intensity noise)^56^, quantization noise^57–59^, galvanometer jitter, mechanical vibrations in the optical system, sample motion, or any other sources of phase instability in the detected signal. Supplementary Section III discusses the effects on the image signal in the space domain, where a coherent average demonstrated a factor of √*N* superior noise reduction efficiency over an incoherent average (Fig. 2a,b). Our results are consistent with previous work in OCT and theoretical trends^51,52^ (see Supplementary Section III for caveats of experimentally achieving the theoretical performance). Namely, noise intensity was reduced by 20 dB (a factor of 100) after a coherent average over *N* = 100 volumes (Fig. 2b, blue circle). Meanwhile, the silicone background intensity was only reduced by 5 dB (Fig. 2b, blue asterisk), suggesting that the silicone medium generated systematic and phase-stable (i.e., not random) backscattered signal.

Here, we additionally investigated coherent-average noise suppression in the spatial-frequency domain (Fig. 2c–e), where the image is a superposition of the detected backscattered signal with a Gaussian magnitude spectrum (when imaged with a Gaussian beam) and the system noise with a uniform magnitude spectrum (circular Gaussian random variable in space). The power spectrum exhibited a dynamic range (DR) in the spatial-frequency domain, as measured from the power at DC to the noise floor (Fig. 2d). A coherent average over *N* = 100 acquisitions resulted in a suppressed noise floor that led to an increase in DR of 20 dB, corresponding to noise reduction by a factor of 100 (Fig. 2d). In other words, the coherent average has revealed phase-stable but low-magnitude backscattered signal at higher spatial frequencies, which were originally below the noise floor in the single-shot image but are now above the suppressed noise floor. Consequently, signal phase remains correlated (i.e., absence of random phase variation) across spatial frequencies corresponding to a larger bandwidth (Fig. 2c,e). The improved correlation of signal phase after reduction in the noise floor can also be understood by considering the impact of SNR on phase noise in phase-sensitive OCT^60,61^. Supplementary Section VII investigates (in simulation) the relations between *N*, DR and phase-correlation limit in the spatial-frequency domain.

### Resolution-enhanced OCT in silicone phantom

**Figure 3:**
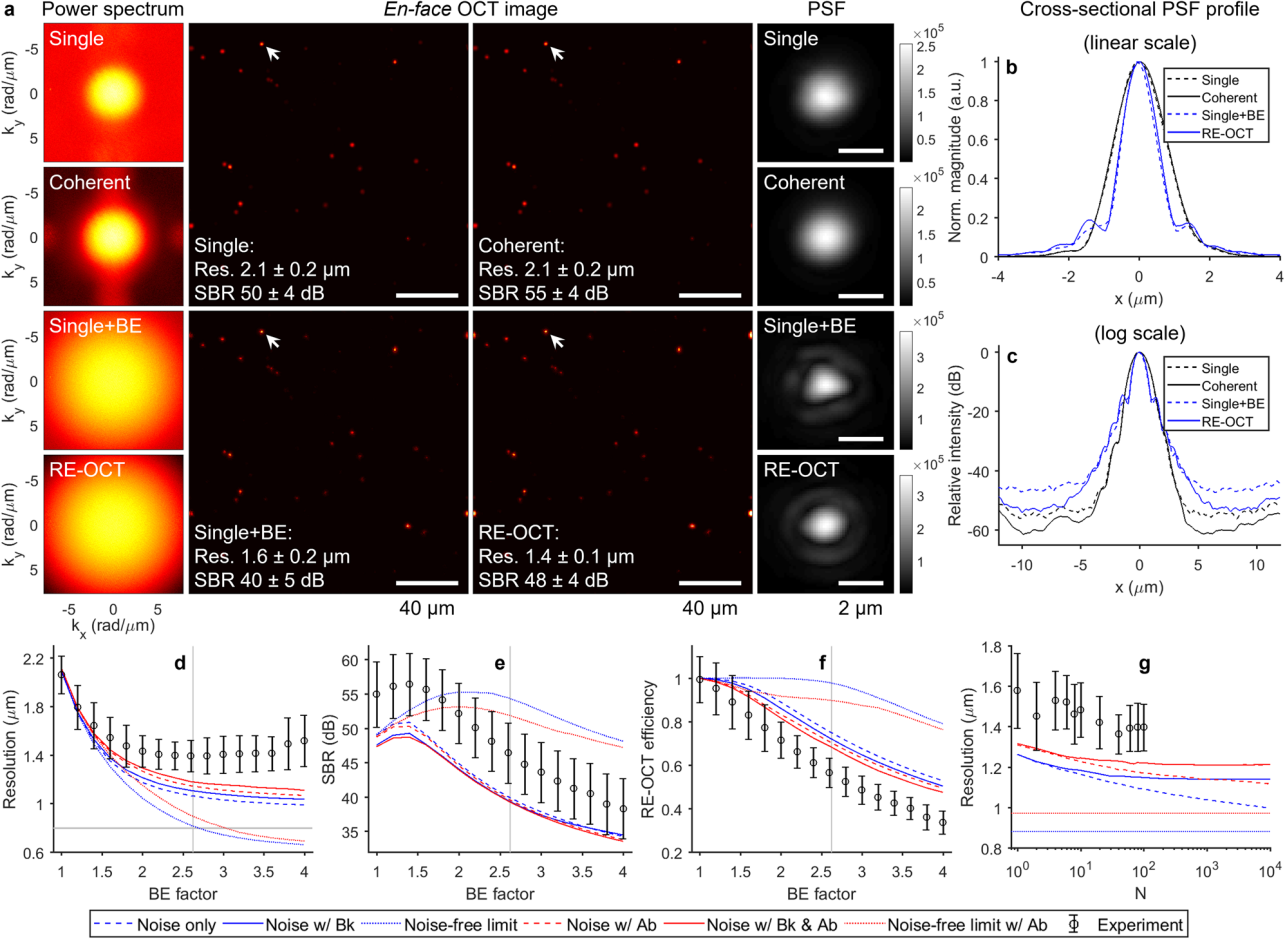
RE-OCT with 100 acquisitions and ×2.4 bandwidth expansion (BE) in silicone phantom. (**a**) Single-shot, coherent-average, BE single-shot, and RE-OCT power spectrums (log scale) and space-domain en face OCT image with zoomed PSF (linear scale). Resolution and SBR represent mean ± standard deviation of measurements from 11 particles. Scale bars, 40 µm (en face image) and 2 µm (zoomed PSF). (**b**,**c**) Cross-sectional profiles of zoomed PSF in (**a**) on peak-normalized linear and log scales. (**d**–**f**) Resolution, SBR, and RE-OCT efficiency as a function of BE factor from experiment and simulations for *N* = 100 of noise-free (dotted), noise only (dashed), and noise with background (solid) conditions, with (red) and without (blue) optical aberrations. Grey vertical lines indicate the Nyquist limit based on spatial sampling of 0.4 µm/pixel. (**g**) Resolution as a function of *N* from experiment and simulations for BE factor of 2.4. Resolution and SBR in (**a**) and data point and error bars for ‘Experiment’ in (**d**–**g**) represent mean ± standard deviation of measurements from 11 particles (see “Methods”). *Bk* background, *Ab* aberrations. See Supplementary Movie 1 for a movie of (**a**–**c**) as increasing BE factor is applied.

Resolution enhancement in silicone phantom using coherent average over 100 acquisitions and a computational BE expansion factor of 2.4× is shown in Fig. 3. (see “Methods” and Supplementary Section IV for a complete description of the RE-OCT reconstruction procedure.) RE-OCT achieved a RE factor of 1.5×, from the traditional aberration-free resolution of 2.1 µm to an enhanced resolution of 1.4 µm (Fig. 3a,b), while the peak signal-to-background ratio (SBR) only marginally decreased from 50 to 48 dB (Fig. 3a,c). (Note that difference in SBR between the single-shot and coherent-averaged images in Fig. 3 is not equivalent to the dB of noise suppression in Fig. 2, see “Methods” for the calculation of SBR). In contrast, although the resolution also improved when computational BE was performed directly on the single-shot image (Fig. 3b), not only was the SBR substantially decreased by 10 dB due to the increased noise floor (Fig. 3c), but the overall quality of the point spread function (PSF) was also degraded (Fig. 3a). This penalty is a result of computational BE indiscriminately amplifying both the backscattered signal and the system noise that dominates at higher spatial frequencies (Fig. 2d,e). Resolution improved at the cost of degraded SBR as a larger BE factor was applied (Fig. 3d,e and Supplementary Movie 1). Noise suppression prior to computational BE was essential in maintaining adequate SBR as well as the quality of the PSF in RE-OCT. However, even with a coherent average over 100 acquisitions, the best achievable resolution from this experiment was limited to 1.4 µm at BE factor of 2.4; applying a larger BE factor only resulted in lower SBR and degraded PSF quality, without further improvement in resolution.

In order to investigate the factors that limit the experimentally achievable resolution enhancement, we performed the RE-OCT procedure on 6 simulated en face planes (3 conditions, each with and without aberrations) with properties representative of the silicone phantom images (Fig. 3d–g) (see Supplementary Section VI for information on the simulated en face planes). Based on Cox and Sheppard’s information capacity framework^4^, the achieved RE factor is expected to be equivalent to the applied BE factor (see Supplementary Section VIII). This relationship holds true for the simulated ideal noise-free condition, in which the RE-OCT efficiency (defined as the ratio RE factor/BE factor) remained 1 up to the Nyquist limit (Fig. 3f, noise-free limit). However, the presence of system noise decreased the RE-OCT efficiency with increasing BE factor (Fig. 3f, noise only). The trends as a function of BE factor for resolution, SBR, and RE-OCT efficiency for the noise-only condition are remarkably consistent with the experimental results (Fig. 3d–f). Furthermore, the presence of scattering signal from the silicone background, in addition to system noise, caused a slight decrease in RE-OCT efficiency relative to the noise-only condition (Fig. 3f, noise with background). These results suggest that system noise is the primary limiting factor in RE-OCT. Indeed, both experiment and simulation showed that superior resolution was achieved with coherent average over larger *N* (i.e., more noise suppression) for a BE factor of 2.4 (Fig. 3g). In addition, optical aberrations degraded resolution, SBR, and RE-OCT efficiency for all simulated conditions (Fig. 3d–g, red). As expected, the simulated condition incorporating all three contributions: system noise, silicone background, and aberrations, most closely matched the experiment.

### Resolution-enhanced OCT in biological samples

**Figure 4:**
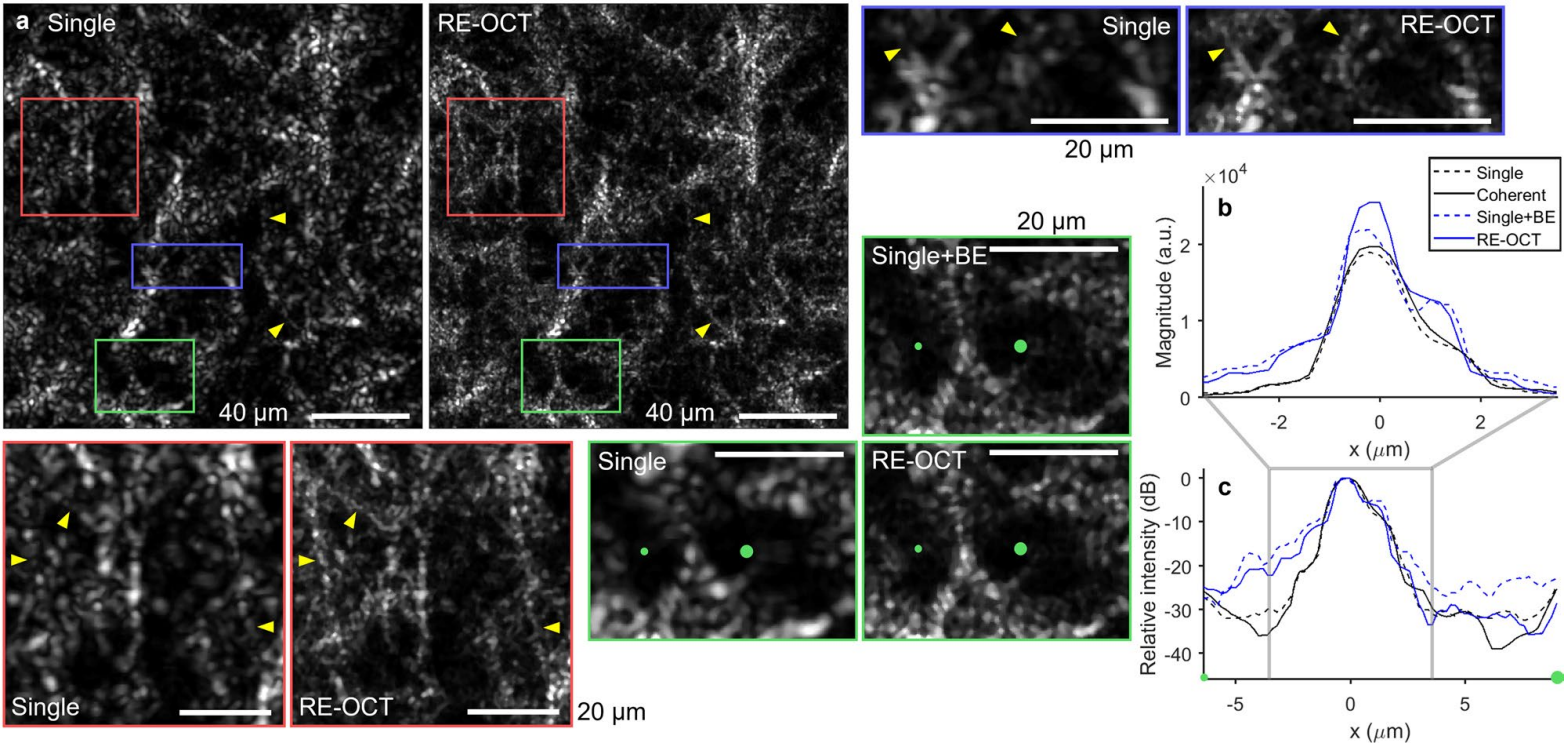
RE-OCT with 100 acquisitions and ×2.0 BE in fibrous collagen gel. (**a**) Single-shot and RE-OCT en face OCT images with zoomed insets regions indicated by boxes. Yellow arrows indicate fine fibre structures that can be more clearly visualized with RE-OCT. Scale bars, 40 µm (full) and 20 µm (zoomed). (**b**,**c**) Cross-sectional profiles of a line connecting from small to larger green dots in the green zoomed insets in (**a**) on linear and peak-normalized log scales. See Supplementary Movie 2 for a movie of (**a**–**c**) as increasing BE factor is applied.

**Figure 5:**
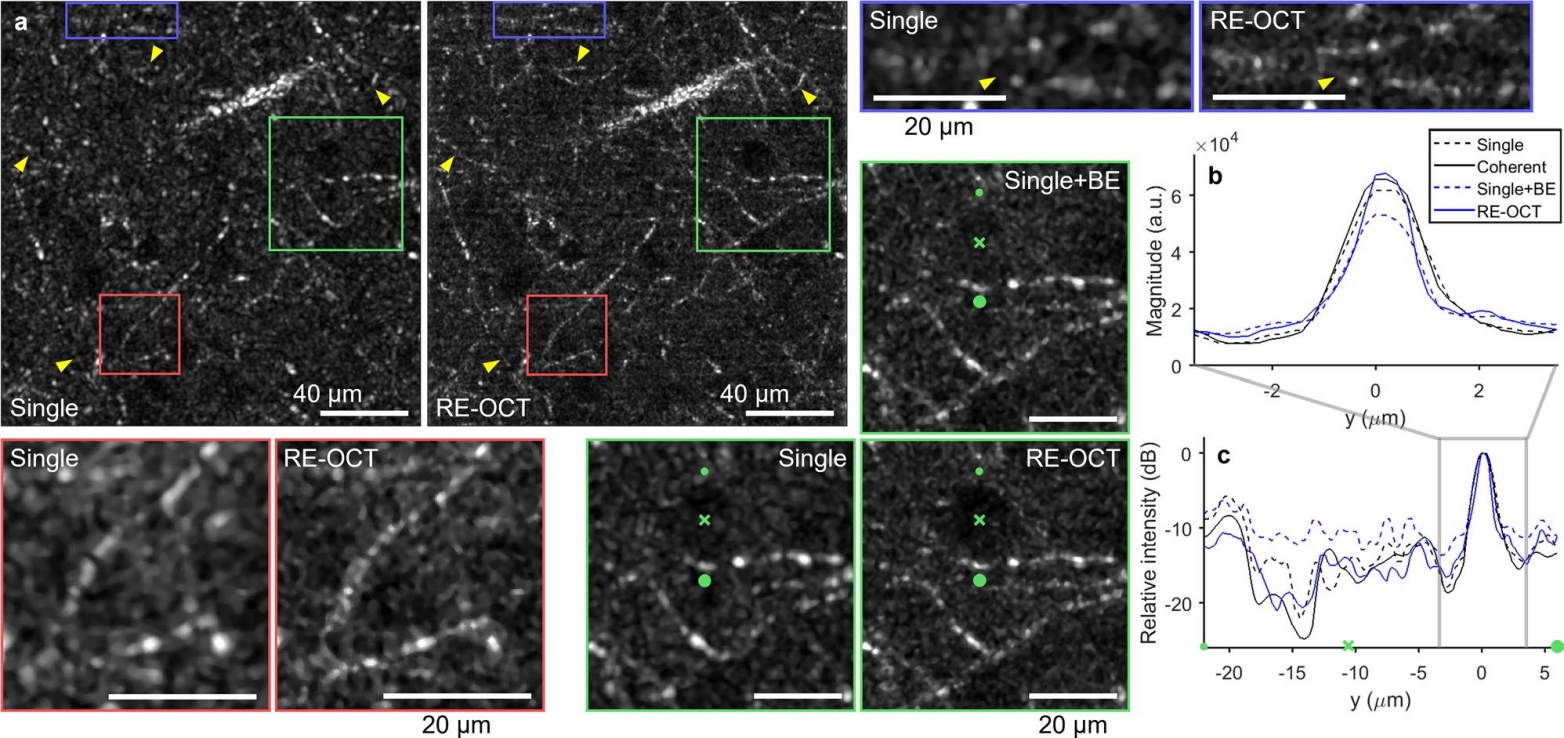
RE-OCT with 100 acquisitions and ×2.0 BE in the cortex of ex vivo mouse brain. (**a**) Single-shot and RE-OCT en face OCT images with zoomed insets regions indicated by boxes. Images were taken in the first cortical layer at approximately 100 µm below surface. Yellow arrows indicate myelinated axonal processes that can be more clearly visualized with RE-OCT. × markers in the green zoomed insets indicate one of the neurons, which appear as darker circles due to weak OCT scattering. Green inset shows that neuron in BE single-shot image was barely discernible due to the SNR penalty without coherent-average noise suppression. Scale bars, 40 µm (full) and 20 µm (zoomed). (**b**,**c**) Cross-sectional profiles of a line connecting from small to large green dots in the green zoomed insets in (**a**) on linear and peak-normalized log scales. The green × marker indicates its corresponding position on the image. See Supplementary Movie 3 for a movie of (**a**–**c**) as increasing BE factor is applied.

We implemented RE-OCT in collagen gel and ex vivo mouse brain, and show the best RE-OCT performances that were achieved, corresponding to BE factor of 2.0 (Figs. 4 and 5). In fibrous collagen gel, RE-OCT enhanced the visualization of the collagen fibre architecture by not only narrowing the width of the collagen fibres, but also increasing the peak signal magnitude of each fibre as a result of the resolution enhancement (Fig. 4a,b). Remarkably, low-contrast fine microstructural features, which were not clearly discernible in the traditional single-shot image due to weak signal, are more apparent in the RE-OCT image owing to the improved localization of signal energy in space (Fig. 4a, yellow arrows). In the BE single-shot image, the narrowing of fibre width can still be observed to a certain extent, but the peak signal magnitude did not improve as much (Fig. 4b). Furthermore, the SBR was degraded more severely in the BE single-shot image due to the amplification of noise without prior noise suppression (Fig. 4c). Computational BE with BE factors larger than 2.0 resulted in degraded SBR without further narrowing of the fibre width (visually assessed) or improvement to the peak signal magnitude (Fig. 4b and Supplementary Movie 2), similar to the degradation observed with BE factors larger than 2.4 in the silicone phantom (Supplementary Movie 1).

In ex vivo fresh mouse brain, RE-OCT enhanced the visualization of myelinated axonal processes, especially the low-contrast features that were less apparent in the traditional single-shot image (Fig. 5a, yellow arrows). Although the narrowing of fibre width could be observed in the BE single-shot image, the peak signal magnitude was degraded without coherent-average noise suppression (Fig. 5b), similar to the effects in collagen gel. Due to the typical fibre thickness of 1–3 µm (which is comparable to the native OCT transverse resolution of 2.1 µm) of myelinated axons^62^, some of the fibre narrowing observed here is not as prominent as in the collagen gel. The SNR penalty of the computational BE procedure is most apparent in the neuron (Fig. 5a, green inset), which produces lower OCT intensity than the surrounding brain tissue. Although the neuron remained visible in the RE-OCT image, the noise level in the BE single-shot image was brought up to that of the backscattered signal from the surrounding brain tissue, causing the neuron to ‘disappear’ into the background (Fig. 5a,c). This emphasizes the importance of coherent averaging in RE-OCT, particularly when weak-scattering structures need to be clearly visualized. Computational BE with BE factors larger than 2.0 resulted in degraded SBR and lower contrast between the neurons and surrounding brain tissue, without further narrowing of the fibre width (visually assessed) or improvement to the peak signal magnitude (Supplementary Movie 3).

### Factors that limit achievable resolution enhancement

We revisit the image signal power and phase in the spatial-frequency domain (Fig. 2c–e) to further understand the role of system noise, background, and optical aberrations on RE-OCT resolution. In the spatial-frequency domain, an increase in system noise will ‘bury’ the signal at higher spatial frequencies first, leading to a reduced DR and an effective reduction in the supported spatial-frequency bandwidth (Fig. 2d); this corresponds to an optimal PSF that is broader in the space domain. More fundamentally, this phenomenon can be explained from the perspective of phase correlation. System noise not only limits the available DR of the spatial-frequency-domain image, but—as a consequence of SNR-limited phase noise—also limits the spatial-frequency bandwidth over which signal phase (associated with any given point scatterer in space) remains correlated (Fig. 2d,e and Supplementary Fig. 7). Phase correlation in the spatial-frequency domain has a direct implication on the spatial resolution of a coherent image—in order to achieve the best localization of signal energy in space, signal at different spatial frequencies must be able to constructively interfere (i.e., be in-phase with each other). Thus, the phase-correlation limit (which is limited by SNR in our experiment) determines how much of the expanded spatial-frequency bandwidth (determined by the BE factor) can support constructive interference and contribute to enhancing the resolution in RE-OCT. Computational BE far beyond the phase-correlation limit only serves to amplify the contribution of phase-decorrelated higher spatial frequencies, which degrades the SBR and the quality of the PSF without further improving the resolution (Fig. 3d–f and Supplementary Movie 1). In this regard, phase correlation in the spatial-frequency domain represents a more fundament (and general) limit to spatial resolution in coherent imaging, where SNR is but one—a predominant one in this case—factor that can disrupt phase correlation.

In contrast to system noise, background is composed of backscattered [single- (SS) and multiple-scattering (MS)] signal from the sample medium (silicone in this case). The spatial-frequency spectrum of the SS background is bandlimited and obeys the imaging bandwidth support of the system (determined by the illumination beam width in our system). Meanwhile, evidence has shown that frequency content of MS background may extend beyond the imaging bandwidth of the system^63^. (However, this work is different from beam-scanned OCT because it performed incoherent imaging using full-field detection.) In either case, background may exhibit uncorrelated phase as opposed to a flat profile of an ideal PSF (Supplementary Fig. 6b) and contribute to the disruption of phase correlation within (SS case) as well as outside (MS case) of the imaging bandwidth. As a result, resolution may be degraded by the presence of background compared to if the medium were completely transparent. In this respect, the role of background on OCT resolution is similar to that of optical aberrations—while aberrations contribute slowly varying phase inside the pupil, background contributes uncorrelated phase that results in the OCT speckle. Importantly, both effects imply that the sample itself may limit the achievable resolution; there can be contribution from sample-induced aberrations in addition to system aberrations, and the degradation of resolution by uncorrelated background phase becomes more severe when the structure of interest has lower SBR (e.g., due to weak scattering from the structure or strong scattering from the medium, or both). Furthermore, both background and aberrations are factors that cannot be mitigated by coherent-average noise suppression.

Beyond system noise, background, and optical aberrations, RE-OCT is also ultimately limited by the spatial-frequency bandwidth support of the optical components (e.g., objective lens), which define the physical aperture outside of which SNR is exactly zero. Computational BE is only effective over the spatial frequencies at which the coherent-averaged spectrum (i.e., the optical transfer function) has *finite* values *above* the suppressed noise floor (which we defined by the phase-correlation limit). In the presented experiments, where the spectrum is a smooth function that under-fills the objective aperture, this limit is imposed by the system noise, which allows RE-OCT to harness additional spatial frequencies via coherent-average noise suppression. However, if the spectrum were to be truncated by the objective aperture (i.e., an aperture-filled system), this limit would be strictly imposed by the physical aperture with no room for further computational BE. That is, RE-OCT can only ‘boost’ the signal that is originally present (i.e., non-zero SNR), but not ‘create’ new signal that did not exist. Retrieving the spatial frequencies beyond the physical aperture of the optical system is beyond the scope of RE-OCT; this would require *analytic continuation*^64^ and could potentially be achieved by some super-resolution and/or deep learning approaches^65,66^.

### Expanded framework of information capacity and resolution in coherent imaging

A fundamental limit to resolution enhancement by RE-OCT is governed by the disruption of phase correlation in the spatial-frequency domain—due to system noise, background, aberrations, and other factors (e.g., sample instability, mechanical vibration, etc.). Among other factors, system noise played the most significant role in our experiments by determining the available DR of the image and the phase-correlation limit in the spatial-frequency domain. Notably, system noise is also the only factor that can be suppressed via coherent averaging in our experiments. Thus, the basis of RE-OCT lies in navigating the trade-off between resolution (in the space domain) and DR (in the spatial-frequency domain) of the image via coherent-average noise suppression and computational BE (Fig. 1a), where DR represents the impact on SNR that manifests in the spatial-frequency domain (Fig. 2d). In Fig. 3a–c, we prioritized resolution enhancement and applied a BE factor of 2.4, which sacrificed more DR than the 20 dB earned with coherent averaging (Supplementary Fig. 8b). Alternatively, we could apply a BE factor of only 1.4 and simultaneously improve both resolution and SBR by a smaller margin (Fig. 3d,e), where the SNR penalty was offset by the increased peak PSF intensity as a by-product of improved localization of the PSF in space (note the maxima in Fig. 3e). The optimal choice of the BE factor is very much dependent on the type of samples and the information that needs to be enhanced for a particular application. Image metrics for the selection of the optimal BE factor could be customized for each specific application.

**Figure 6:**
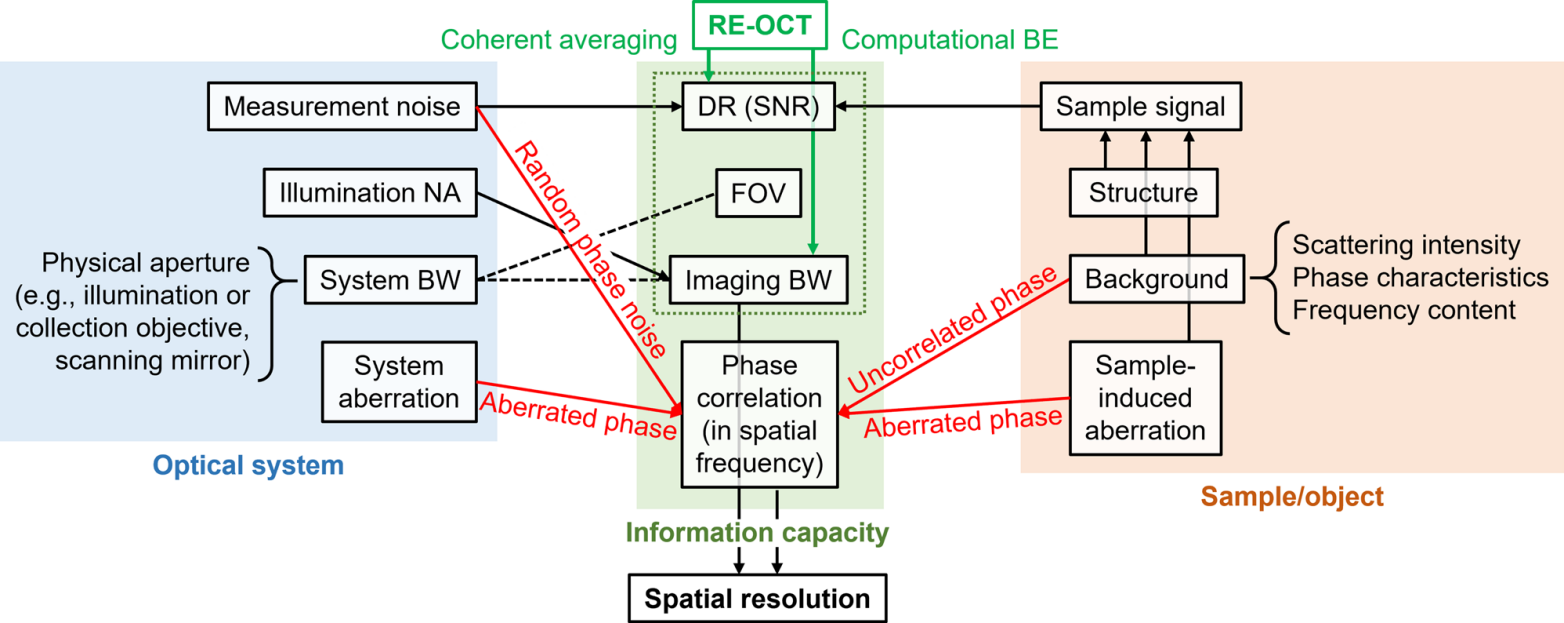
Expanded framework of information capacity and resolution in coherent imaging. Factors that influence elements of the expanded information capacity and ultimately affect resolution in coherent imaging. Phase correlation in the spatial-frequency domain is an important addition to the existing framework of information capacity (green dotted box)^4^. Red arrows denote factors that can disrupt phase correlation in the spatial-frequency domain. Green arrows indicate the role of RE-OCT: enhancing DR in the spatial frequency domain via coherent-average noise suppression, then, expanding imaging bandwidth via computation BE. For the sake of simplicity, the depicted framework omits the temporal components and only considers information in the spatial dimensions. Supplementary Section VII discusses the relationship between system and imaging bandwidths, illumination NA, and FOV. BW, bandwidth.

In order to reconcile the predictions of information capacity and our experimental RE-OCT results, we propose an expanded framework of information capacity and resolution in coherent imaging (Fig. 6). The expanded framework emphasizes phase correlation in the spatial-frequency domain (in addition to SNR, FOV and spatial-frequency bandwidth in Cox and Sheppard’s framework^4^) as an important facet of the information capacity of a coherent imaging system. In theory, resolution is governed by the imaging bandwidth of the optical system. In practice, however, phase-correlation limit in the spatial-frequency domain (Fig. 2e and Supplementary Fig. 7) must also be considered when determining the best achievable resolution. Supplementary Section VIII computes the resolution enhancement that is theoretically supported by Cox and Sheppard’s information capacity framework^4^, and exemplifies the additional practical limit imposed by the SNR-limited phase-correlation limit (Supplementary Fig. 8a). Importantly, any factors—whether associated with the optical system or the sample itself—that can disrupt phase correlation in the spatial-frequency domain may prevent the optimal resolution (determined by the bandwidth support of the optical system) from being experimentally realized. We postulate that the *theorem of invariance of information capacity* (Eq. 1) could, in principle, be restated in term of phase correlation rather than SNR, however, this theoretical advance is beyond the scope of this paper. By extension, in a time-dependent system whose information capacity includes the time-bandwidth product^4^, the temporal resolution of such a system would also be subjected to the correlation of phase in the temporal-frequency domain.

## Discussion

Beam-scanning is by far the most common mode of acquisition in OCT. Most beam-scanned OCT systems utilize a Gaussian illumination beam that under-fills the physical aperture limit (e.g., objective lens) in the optical system. This under-filling is required for the widely used telecentric scanning scheme (as opposed to pivoting at the objective aperture), which is implemented to minimize distortions due to coherence gate curvature^67^. (Although ophthalmic OCT systems do not implement telecentric scanning, the typical beam diameter of 1.2 mm in standard clinical systems^68^ still under-fills the physical aperture of typical pupil diameters of adult eyes^69^). RE-OCT exploits this ubiquitous design feature in OCT systems to enable resolution enhancement beyond the aberration-free limit achieved by existing aberration correction approaches. In beam-scanned OCT, the aberration-free resolution limit is determined by a combination of the illumination beam width and the objective lens (i.e., the smaller of the Gaussian beam NA or the objective lens NA, see Supplementary Section II); for a telecentric-scanning OCT, the Gaussian beam NA is typically much smaller. Higher resolution is traditionally achieved by increasing the beam width or simply switching to a higher-NA objective (a standard practice in optical microscopy). RE-OCT, on the other hand, offers the flexibility to enhance transverse resolution beyond the aberration-free limit, using a simple computational procedure, without requiring any modification to the optical system or specialized acquisition schemes in existing aperture synthesis techniques. This gives RE-OCT a remarkable potential to have a widespread impact as it can be readily implemented on most OCT systems in the world and immediately unlock information that is beyond the current imaging capability of the system.

Phase stability is vital for achieving optimal performance in RE-OCT, as is the case in other phase-sensitive techniques in coherent imaging such as CAO and OCT angiography, which poses potential challenges for biological (e.g., live-cell imaging) and clinical (e.g., in vivo imaging) applications. In the case of RE-OCT, efficient earning of SNR via coherent-average noise suppression is contingent upon the backscattered signal from multiple acquisitions being phase-registered to each other. Our experiments in ex vivo fresh mouse brain (Fig. 5) required an additional image registration procedure (Supplementary Section V) to achieve phase registration before computing the coherent average. Others in OCT have also developed image registration and phase correction methods that successfully enabled phase-sensitive processing in biological samples, including for in vivo settings^27,70–72^. Furthermore, advances in high-speed imaging have enabled OCT imaging at MHz-rate^73^, combatting motion artefacts and supporting phase-sensitive imaging in vivo^29–31,52,74–76^. RE-OCT may be particularly attractive for high-speed systems, some of which already incorporate coherent or incoherent averaging^52,74,75^, where the RE-OCT procedure can be easily integrated into the existing imaging workflow with minimal additional effort and imaging time. Thus, RE-OCT can draw from existing and emerging solutions in the field to address the challenges associated with achieving the required phase stability for biological and clinical applications, including in vivo imaging.

Beyond the potential applicability of RE-OCT for OCT systems worldwide, the concept that noise suppression (improved SNR) can be harnessed for resolution enhancement—where efficient noise suppression is key to ‘purchasing’ greater resolution enhancement—has broad implications for not only OCT but also optical microscopy and imaging science. Traditionally, averaging and noise suppression have been associated with the improvement of image contrast or SBR in optical microscopy. However, the *theorem of invariance of information capacity* suggests that there are opportunities to exploit the information gained via noise suppression in other facets of optical imaging. RE-OCT applies this concept to improve resolution through an exchange of information between spatial-frequency bandwidth and SNR.

Furthermore, the expanded framework of information capacity and resolution in coherent imaging presented here is broadly relevant and contributes to the theory governing coherent image formation. This includes coherent imaging with an aperture-filled system (e.g., full-field OCT) even though RE-OCT is only applicable when the physical aperture is under-filled. For instance, an image of a sample that generates particularly weak signal may have a very limited DR in the spatial-frequency domain, such that the SNR-limited phase-correlation limit is *smaller* than the imaging bandwidth of the optical system. Resolution would be limited by the available DR as opposed to the illumination NA (in an under-filled system) or the objective NA (in an aperture-filled system) in such a low-SNR scenario. Alternatively, the detected backscattered signal may be well above the noise floor, but both the object and the surrounding medium contribute comparable signal strength such that the DR spanned by the SS signal level in the sample is low. The disruption of phase correlation by the SS and MS background could limit the ability to resolve the object in such a low-DR scenario. By extension, physically increasing the image bandwidth of an optical system (e.g., by using a higher-NA objective or increasing the illumination beam width) would yield the optimal improvement in resolution *only if* the acquired image had sufficient DR in the spatial-frequency domain to support phase correlation over the increased bandwidth. Thus, our expanded framework highlights important practical considerations (associated with both the optical system and the sample/object) for resolution in all forms of coherent imaging.

Future development may combine RE-OCT with aberration-diverse OCT^55^ in order to suppress both the system noise and the MS background. Additionally, RE-OCT may be advantageous for imaging transversely isotropic structures (i.e., spatially *invariant* along a given spatial dimension) such as aligned muscle fibres or organized collagen fibrils in tendon and cartilage. This could allow for bandwidth along both the temporal and the invariant spatial dimension to be sacrificed to further enhance the resolution along the orthogonal spatial dimension^3^ Furthermore, the same RE-OCT approach can be applied to enhance the axial resolution of an SD-OCT system by ‘boosting’ the tails of the source spectrum within the spectrometer bandwidth, provided that the broadband light source ‘under-fills’ the spectrometer.

## Conclusion

RE-OCT is an approach that offers the flexibility to enhance resolution in beam-scanned OCT beyond the aberration-free resolution limit of the optical system. RE-OCT can be readily implemented on most OCT systems in the world without requiring any modification to the optical system. Based on the *theorem of invariance of information capacity*, RE-OCT navigates the information exchange between resolution and SNR by ‘earning’ SNR via coherent-average noise suppression, in order to ‘purchase’ superior resolution via computational BE. Coherent averaging was shown to increase DR in the transverse spatial-frequency domain, and for the first time, has been harnessed for resolution enhancement in OCT. In silicone phantom, RE-OCT achieved a resolution improvement of 1.5× (NA increase of 0.2 to 0.3), while maintaining comparable SBR to the traditional single-shot image. In collagen gel and ex vivo mouse brain, RE-OCT significantly enhanced the visualization of fine microstructural features, including low-contrast features that were otherwise obscured in the traditional OCT image. We found that the phase-correlation limit represents an additional practical limit to the effective spatial-frequency bandwidth support of a coherent imaging system that can be more restrictive that the theoretical limit imposed by the existing theory of information capacity^4^. Based upon these insights, we presented an expanded framework of information capacity and resolution in coherent imaging to incorporate these factors. This framework emphasizes the fundamental role of phase correlation, which contributes important implications to the theory of coherent imaging.

## Methods

### Optical system

The optical system was a standard telecentric beam-scanned spectral-domain (SD)-OCT system (Supplementary Fig. 2a). The SD-OCT system was sourced by a broadband superluminescent diode with a central wavelength of 850 nm and a bandwidth of 120 nm (Superlum, M-T-850-HP-I). Spectral data was detected by a spectrometer with a bandwidth of 180 nm (Wasatch Photonics, Cobra 800) and a 2048-pixel line-scan camera (e2v, Octopus). The sample arm utilized a double-pass illumination/collection configuration with an inverted 20× microscope objective with an NA of 0.45 (Olympus, LCPLN20XIR). Telecentric beam-scanning was accomplished with a 2-axis galvanometer and a zero-magnification telescope, which imaged the galvanometer to the back focal plane of the objective. The illumination beam diameter was ~ 2–3 mm, which under-filled the objective back aperture diameter of 8.1 mm. The native transverse resolution was 2.1 µm at the focal plane and the axial resolution was 1.9 µm in air. The system sensitivity was ~ 90 dB at the implemented acquisition rate (see RE-OCT image acquisition procedure) with a fall-off of − 5 dB/mm. The system was controlled by a custom-built acquisition software in LabVIEW 2014 (https://www.ni.com/en-us/shop/labview.html).

### Sample preparation

All samples were prepared in glass coverslip-bottomed petri dishes, where the OCT beam interrogated the sample from the bottom through the coverslip (Supplementary Fig. 2c). The “noise image” for measuring the system noise in Fig. 2b,d was acquired by imaging the empty sample dish (Supplementary Fig. 2c).

Silicone phantom (Figs. 1 and 2) was prepared with a mixture of polydimethylsiloxane (PDMS) fluid (Clearco Product, PSF-50cst) and 2-part RTV silicone (Momentive Performance Materials, RTV-615 CLEAR 1#) at a weight ratio of 100:10:1 PDMS to RTV A to RTV B. Titanium dioxide particles with diameter of 0.5 µm were dispersed as scattering particles. Silicone mixture was baked at 70 °C for at least 8 h to complete the polymerization process. The sample was stored room temperature, where the temperature was allowed to stabilize, prior to imaging.

Collagen gel (Fig. 4) was prepared with type I collagen (Corning, Collagen I, rat tail) at a final collagen concentration of 2.0 mg/mL. Collagen was polymerized at 4 °C for 15 min., 20 °C for 15 min., and finally 37 °C for 15 min. to promote formation of heterogeneous fibre architecture with thick collagen fibres^77^. The sample was removed from incubation 1–2 h before imaging to allow the temperature to stabilize at room temperature.

Ex vivo mouse brain (Fig. 5) was harvested post-mortem from a C57BL/6 mouse. Euthanasia was induced by an intraperitoneal injection of pentobarbitol (150 μL of 39 mg/mL solution in saline) and then perfused via intracardiac puncture with 30-mL phosphate-buffered saline (PBS) at 4 °C. The harvested brain was stored in PBS at 4 °C before embedded in 1% agarose (Sigma-Aldrich, Agarose, low gelling temperature) in the sample dish without fixation. The sample was kept at room temperature for 1–2 h to allow the temperature to stabilize at room temperature before imaging. All animal procedures were approved by the Cornell Institutional Animal Care and Use Committee and were performed under the guidance of the Cornell Center for Animal Resources and Education. The study was carried out in compliance with the ARRIVE guidelines.

### RE-OCT image acquisition procedure

Images were acquired in CM mode, where 3D OCT volumes were acquired successively to allow sufficient decorrelation of noise (see Supplementary Section III). Each volume was acquired with a line scan rate of 70 kHz, an exposure time of 10 µs, and a transverse spatial sampling of 0.4 µm/pixel. In order to maximize the dynamic range spanned by the signal from the sample, image was acquired in the *conjugated configuration* by adjusting the reference arm such that the coverslip-bottom of the sample dish was positioned at larger pixel depths near the bottom of the B-scan (see Supplementary Section II).

### RE-OCT image reconstruction procedure

RE-OCT image reconstruction from the acquired CM-mode volumes followed the procedure described in Supplementary Section IV. Briefly, space-domain OCT volumes were obtained from the raw tomograms via standard OCT image reconstruction, then, corrected for defocus via computational image formation procedures based on previously described methods^78^. For ex vivo mouse brain, an additional image registration procedure was required to correct bulk sample shift and phase drift in order to ensure that backscattered signal was spatially- and phase-registered across CM-mode volumes, as described in Supplementary Section V. Then, coherent average across processed OCT volumes was computed and its magnitude spectrum was obtained from the 2D transverse Fourier transform. A BE mask was computed from the magnitude spectrum at a given BE factor and applied to the coherent-average OCT volume in the transverse spatial-frequency domain. Finally, the BE spectrum was zero-padded to upsample (in space) before returning to the space domain. The spatial upsampling was implemented to facilitate resolution measurement via curve-fitting to the PSF. All RE-OCT image reconstruction and subsequent image processing was performed in MATLAB R2017a. All OCT images shown has undergone defocus correction and represent the traditional aberration-free imaging capability before computational BE.

### Calculations of OCT intensity

The OCT intensity of the scattering particles, silicone background, and noise in Fig. 2b were computed as follows. Scattering particle intensity was obtained from the 99th percentile of the OCT scattering intensity (i.e., square of OCT magnitude) of the space-domain image at the focal plane. Silicone background intensity was obtained from the median of the OCT scattering intensity of the particle-removed space-domain image at the focal plane. The scattering particles were removed from the en face image via magnitude thresholding followed by a dilatation of the binary mask. Noise intensity was obtained from the standard deviation of the OCT scattering intensity of the “noise image” at the same pixel depth as the focal plane of the silicone phantom image. The “noise image” was obtained by imaging an empty blank sample dish (Supplementary Fig. 2c), placing the coverslip at the same pixel depth as in the silicone phantom.

### Calculation of dynamic range and phase-correlation limit

The dynamic range in Fig. 2d and Supplementary Fig. 7 was computed as follows. First, the noise power spectrum was obtained from the square of the magnitude of the 2D transverse Fourier transform of the “noise image” at the same pixel depth as the focal plane of the silicone phantom image. Then, the relative noise power was computed w.r.t. the signal power at DC (i.e., *k*_*r*_ = 0 rad/µm) of the silicone phantom image at the focal plane. DR value in decibels was obtained from the mean of the relative noise power spectrum (uniformly distributed) across the entire transverse spatial-frequency domain.

The phase-correlation limit in Fig. 2e and Supplementary Fig. 7 was computed as follows. First, a window of size 243 × 243 pixels centred on a single scattering particle was cropped from the silicone phantom image at the focal plane. Then, the phase spectrum of the PSF was obtained from the angle of the 2D transverse Fourier transform of the window-out region. Next, local standard deviation of the phase spectrum was computed over a sliding kernel of size 3 × 3 pixels to obtain the “phase-decorrelation spectrum”. The “phase-decorrelation spectrum” was divided into spatial-frequency bins, ranging from *k*_*r*_ = 0 rad/µm to *k*_*r*_ = 7.85 rad/µm (the Nyquist limit) at bin width of 0.1 rad/µm. Phase-correlation limit was obtained from the *k*_*r*_ value at the centre of the bin at which the mean of the “phase-decorrelation spectrum” exceeded 0.2 rad.

### Measurements of resolution and SBR

Resolution and SBR values in Fig. 3a,d–f were obtained from the Gaussian curve-fit to the PSFs (i.e., scattering particles) located at the focal plane. First, maximum intensity projection across 3 pixel-depths about the focal plane was computed from the OCT magnitude image. Scattering particles at the focal plane with peak magnitude > 5 × 10^4^ were manually identified and the PSF images (a window of size 73 × 73 pixels centred on each particle) were cropped out. Then, a total of 32 radial cross-sectional profiles of the PSF images (i.e., 1D PSF profiles at 32 different angular cross sections) were extracted for linear least-square curve fitting to a 1D Gaussian function. The fit parameters from the 32 cross-sectional profiles were averaged to obtain the peak magnitude and full width at half-maximum (FWHM) of each particle. At this stage, particles with FWHM > 2.4 µm measured from the coherent-average OCT volume (i.e., noise-suppressed but not bandwidth-expanded) were excluded for being either air bubbles or aggregates of multiple particles. A total of 11 particles remained after the exclusion.

Resolution was obtained directly from the mean FWHM of the 11 remaining particles. SBR was obtained from the mean “peak SBR” of the 11 particles. The “peak SBR” in decibels of each particle was computed from the ratio of the peak PSF intensity (square of peak magnitude from the Gaussian fits described above) to the silicone background intensity (computed as described in Calculations of OCT intensity).

## Supplementary Information

Supplementary Information.

Supplementary Video 1.

Supplementary Video 2.

Supplementary Video 3.

## Data Availability

Data underlying the results presented in this paper are not publicly available at this time but may be obtained from the authors upon reasonable request.
