# Supplementary material for: Resolution-enhanced OCT and expanded framework of information capacity and resolution in coherent imaging
Source: Sci Rep. 2021 Oct 15;11:20541. doi: 10.1038/s41598-021-99889-3 (PMC8521598; doi:10.1038/s41598-021-99889-3)
Supplement: Supplementary file 1 — Supplementary Information. [file 41598_2021_99889_MOESM1_ESM.docx]

**Resolution-enhanced OCT and expanded framework of information capacity and resolution in coherent imaging: supplementary information**

Nichaluk Leartprapun^1^ and Steven G. Adie^1,*^

^1^Nancy E. and Peter C. Meinig School of Biomedical Engineering, Cornell University, Ithaca, New York 14853, USA

*Correspondence should be addressed to sga42@cornell.edu

**Contents**

Supplementary Section I: Information capacity and resolution enhancement via exchange of information

Supplementary Section II: System diagram, conjugated imaging configuration, and telecentric scanning

Supplementary Section III: Signal averaging in the space domain and caveats for achieving theoretical noise suppression performance

Supplementary Section IV: RE-OCT reconstruction procedure

Supplementary Section V: Image registration procedure for *ex vivo* mouse brain

Supplementary Section VI: Simulation of RE-OCT in silicone phantom

Supplementary Section VII: Noise, dynamic range, and phase correlation in the spatial-frequency domain

Supplementary Section VIII: Fundamental limits to resolution enhancement in RE-OCT

Supplementary Movie 1 Caption

Supplementary Movie 2 Caption

Supplementary Movie 3 Caption

**Supplementary Section I: Information capacity and resolution enhancement via exchange of information**

Cox and Sheppard derived an expression for the information capacity (in bits) of an optical system^1,2^ (Eq. 1), which we restate here:

$C=\left(2L_{x}B_{x}+1\right)\left(2L_{y}B_{y}+1\right)\left(2L_{z}B_{z}+1\right)\left(2TB_{T}+1\right)\log_{2}\left(1+s/n\right)$, (S1)

where $L$, $T$, and $B$ denote the spatial field-of-view (FOV), temporal duration, and bandwidth in the associated dimension, respectively. The first three terms represent the space-bandwidth product (SBP) along the three spatial dimensions. The fourth term represents the time-bandwidth product (TBP). The last term represents the signal-to-noise ratio (SNR) in the logarithmic scale, where $s$ and $n$ denote the average signal power and additive noise power, respectively. For an imaging configuration where SBP, TBP and SNR >> 1, the information capacity of a single acquired volume simplifies to:

$C_{\text{single}}=\left(2L_{x}B_{x}\right)\left(2L_{y}B_{y}\right)\left(2L_{z}B_{z}\right)\left(2TB_{T}\right)\log_{2}\left(s/n_{\text{single}}\right)$. (S2)

Consider repeatedly acquiring an image of a ‘static’ object *N* times. Since an object that is known a priori to be invariant in time possesses effectively zero temporal bandwidth (i.e., *B_T_* = 0), the factor of *N* increase in *T* does not result in an increase in the information capacity—the gain in the temporal information is essentially redundant. In other words, although *N* repeated acquisitions provide the capacity to support *N* times more information, no additional information is directly derived from each subsequent image—essentially a replica of the previous image in a ‘static’ sample—to actually ‘occupy’ this expanded capacity. Now consider coherently averaging the *N* acquired images of the ‘static’ object. This process “encodes” the originally redundant increase in *T* in the finite SNR term in the new coherent-averaged system. In other words, if the object is known *a priori* to be invariant in time, the object can be successively imaged *N* times (increasing $T$ by a factor of *N*) and the OCT datasets coherently averaged (reducing $B_{T}$ by factor of *N*), which keeps the time-bandwidth product unchanged but suppresses the system noise by a factor of 1/*N*—creating a system with larger information capacity resulting from the enhanced SNR. The gain in information capacity via the SNR term of the coherent-average volume relative to the single-shot volume is given by:

$C_{\text{avg}}=\left(2L_{x}B_{x}\right)\left(2L_{y}B_{y}\right)\left(2L_{z}B_{z}\right)\left(2TB_{T}\right)\log_{2}\left(s/n_{\text{avg}}\right)\text{ ; }n_{\text{avg}}=n_{\text{single}}/N$ (S3)

$\frac{C_{\text{avg}}}{C_{\text{single}}}=\frac{\log_{2}\left(s/n_{\text{avg}}\right)}{\log_{2}\left(s/n_{\text{single}}\right)}=1+\frac{\log_{2}\left(N\right)}{\log_{2}\left(s/n_{\text{single}}\right)}$ (S4)

In this case, the redundant *N* times increase in *T* is instead transformed into a finite increase in SNR—a lossy transformation, since the information gain in Eq. S4 is less than *N*—that ‘occupies’ the expanded capacity. Based on the *theorem of invariance of information capacity*, the extra information capacity earned via coherent-average noise suppression can be distributed to the SBP terms (e.g., by equally increasing $B_{x}$ and $B_{y}$) in order to enhance the resolution along those dimensions. According to the information capacity $C_{\text{avg}}$ given in Eq. S3, the allowable bandwidth expansion (BE) factor, $\varepsilon_{\text{BE}}$, that can be achieved without penalty to the SNR w.r.t. the original single-shot volume is given by:

$\varepsilon_{\text{BE}}^{2}=\frac{B_{x,\text{RE}}B_{y,\text{RE}}}{B_{x}B_{y}}=\frac{\log_{2}\left(s/n_{\text{avg}}\right)}{\log_{2}\left(s/n_{\text{single}}\right)}$, (S5)

where $B_{x,\text{RE}}$, and $B_{y,\text{RE}}$ are the expanded spatial-frequency bandwidths in the $x$ and $y$ dimensions, respectively. Note that it is the *square* of BE factor that scales with the factor of log-scale SNR gain (RHS of Eq. S5) because the extra SNR is equally distributed between the *two* spatial dimensions in this scenario. The resolution enhancement (RE) factor,$\varepsilon_{\text{RE}}$, supported by this information exchange process is then given by:

$\varepsilon_{\text{RE}}=\frac{\text{Res}}{\text{Res}_{\text{RE}}}=\frac{B_{x,\text{RE}}}{B_{x}}=\frac{B_{y,\text{RE}}}{B_{y}}=\varepsilon_{\text{BE}}$, (S6)

where $\text{Res}$ and $\text{Res}_{\text{RE}}$denote the original and the enhanced transverse resolution, respectively. Note that since the $\varepsilon_{\text{RE}}$ is a function of the *ratio* of log-scale SNR, the base of the log does not influence the supported resolution enhancement (i.e., although log base 2 for SNR and information capacity in bits is used in Eq. S1–5, the same $\varepsilon_{\text{RE}}$ prediction could be obtained with log base 10 for SNR in dB scale). The factor of log-scale SNR gain and the corresponding supported RE factor are shown as a function of *N* in Supplementary Fig. 1. Supplementary Section VIII deals with more general cases where an arbitrary amount of SNR, not limited to the amount earned via coherent average, may be sacrificed to enhance resolution, as done in the silicone phantom experiment (Fig. 2).


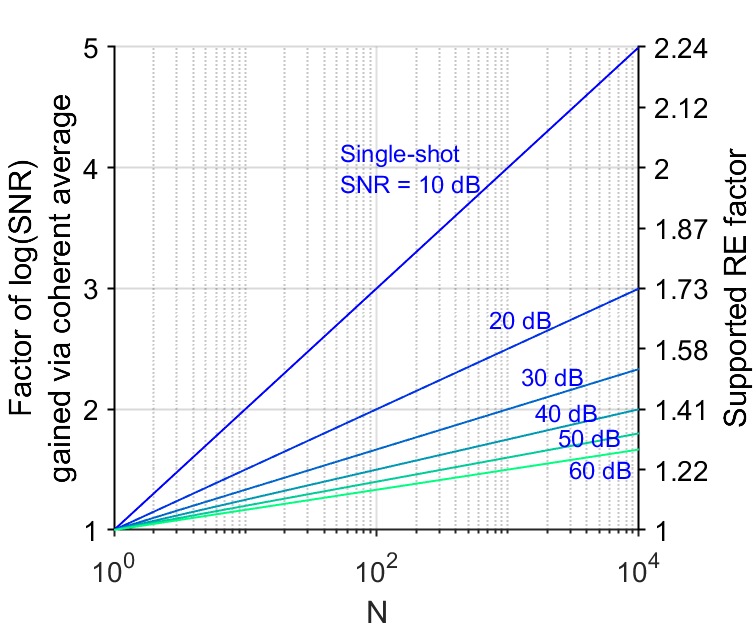


**Supplementary Figure 1 Resolution enhancement supported by coherent-average noise suppression.** Gain in logarithmic-scale SNR and the corresponding RE factor supported by the invariance of information capacity as a function of *N*. Curves are shown for different original SNR of the single-shot volume. See Supplementary Section VIII for general cases where more SNR than earned may be sacrificed to enhance resolution.

**Supplementary Section II: System diagram, conjugated imaging configuration, and telecentric scanning**

The spectral-domain (SD)-OCT system diagram is shown in Supplementary Fig. 2a. Details of the optical components are provided in Methods. Imaging was performed in an inverted setup where the OCT beam interrogated the sample through the coverslip-bottom of the sample dish. The coverslip is essential for phase registration of individual OCT volumes (see Supplementary Section IV).


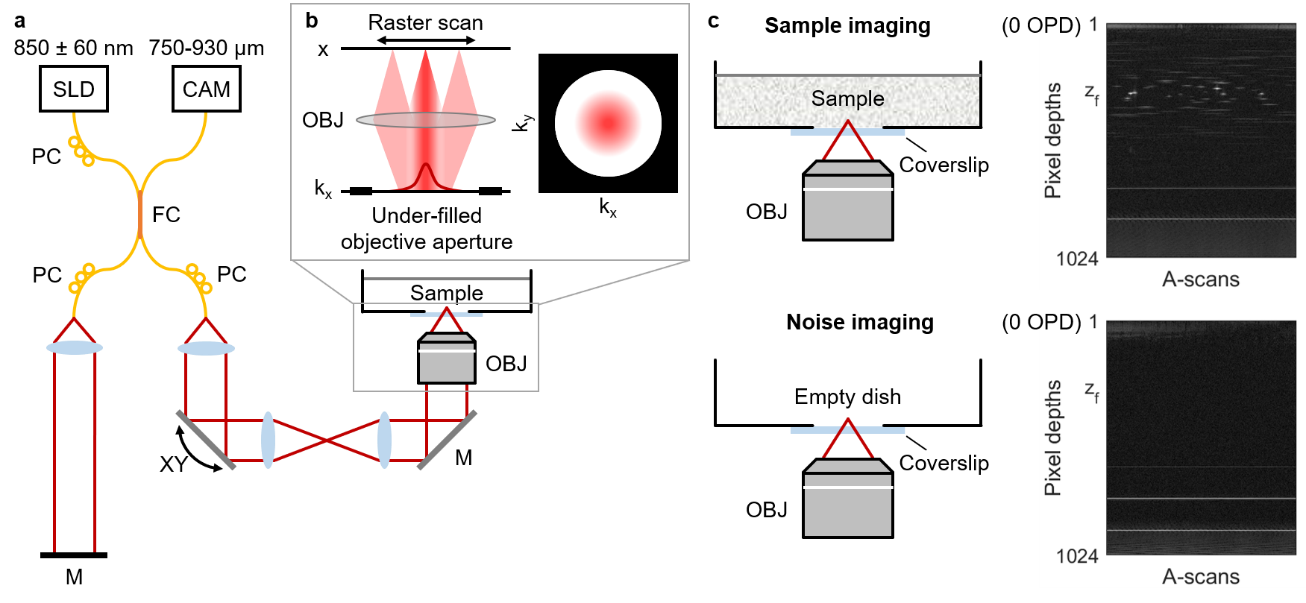


**Supplementary Figure 2 Experimental setup. a**, SD-OCT system diagram. SLD, superluminescent diode. CAM, spectrometer and line-scan camera. PC, polarization controller. FC, 50/50 fiber coupler. M, mirror. XY, 2-axis galvanometer mirrors. OBJ, objective lens. **b**, Under-filling of objective aperture for telecentric scanning. **c**, Sample configuration and example cross-sectional OCT image demonstrating imaging in the conjugated configuration. *z*_f_ indicates the focal plane of the sample image.

In this setup, the coverslip would appear closer to the 0 optical path difference (OPD) if it were imaged with the reference mirror positioned for imaging in the *traditional imaging configuration*, causing the signal at the camera to be dominated by the strong reflection at the glass-air and glass-sample interfaces of the coverslip. In other words, the actual signal of interest from the sample would only be able to occupy a small portion of the available dynamic range of the camera as the reference arm power was adjusted to avoid saturation at the coverslip interfaces. In order to maximize the dynamic range coverage of the signal from the sample, imaging was actually performed in the *conjugated configuration* where the reference mirror position was adjusted to wrap the coverslip toward higher pixel depths and placed the focal plane inside the sample closer to the 0 OPD (Supplementary Fig. 2c). This configuration exploited the spectrometer roll-off to maximize signal from the focal plane while suppressing strong reflection from the coverslip.

The SD-OCT system in Supplementary Fig. 2a utilized telecentric beam-scanning to acquire 3D tomograms. Telecentric scanning is the preferred beam-scanning scheme in OCT in order to minimize coherence gate curvature. In such a system, the physical aperture of the objective lens is underfilled by the illumination beam in order to allow for telecentric scanning—by imaging the galvanometer mirror to the objective back focal plane—‘across’ the objective lens (Supplementary Fig. 2b). Thus, the numerical aperture (NA) of the system is determined by the width of the illumination Gaussian beam (typically the 1/*e*^2^ width) rather than the objective aperture diameter, which sets a physical limit to the system bandwidth. The transverse resolution of the system can often be adjusted (to a certain extent) by changing the width of the illumination beam (by modifying the collimating or telescope optics) without switching the objective lens. However, there is a tradeoff between beam width and the FOV that can be supported without clipping the beam. Alternatively, an objective lens with a higher NA (e.g., shorter focal length) can be used to achieve better transverse resolution. However, there is also a tradeoff between the physical system bandwidth (i.e., objective aperture) and the supported FOV and working distance in a typical microscope.

**Supplementary Section III: Signal averaging in the space domain and caveats for achieving theoretical noise suppression performance**

Figure 1b shows the signal intensity of the scattering particles, silicone background, and noise after incoherent and coherent average over N acquisitions. Signal intensity (i.e., OCT magnitude-square) of the scattering particles remained unchanged in both incoherent- and coherent-average images (Fig. 1b, triangle), indicating that the signal was dominated by phase-stable backscattering from the particles. In contrast, signal intensity in the silicone background was reduced by coherent average before stabilizing after *N* > 10 acquisitions (Fig. 1b, asterisk), suggesting that the silicone medium generated phase-stable in time (albeit low-magnitude) backscattering signal that was initially ‘hidden’ by noise and later revealed by coherent average. Faint scattering signal from the silicone background can be at the focal plane of the coherent-average, but not the traditional single-shot image (Fig. 1a, cross-sectional image). Meanwhile, the incoherent average failed to suppress the average background intensity (Fig. 1a,b). Noise images of an empty sample dish were acquired to quantify the system noise (Supplementary Fig. 2c), defined as the standard deviation of signal intensity across the transverse FOV. Both incoherent and coherent averages suppressed the noise, but the coherent average was more efficient (Fig. 1b, circle). Noise reduction followed the theoretical trends of 1/√*N* and 1/*N* for incoherent and coherent averages, respectively^3,4^, demonstrating a factor of √*N* superior efficiency in noise suppression by coherent over incoherent average.

The theoretical coherent-average noise suppression efficiency with a factor of 1/*N* under is based on the premise that different realizations of noise, a circularly symmetric complex random variable, are uncorrelated. For the system employed in this study, this condition was achieved when successive OCT volumes, acquired with a CM-mode acquisition scheme (see Methods), were coherently averaged (Fig. 1b and Supplementary Fig. 3a, solid lines). However, noise suppression failed to reach the theoretical 1/*N* efficiency when successive B-scans, acquired with a BM-mode acquisition scheme (i.e., acquiring multiple B-scans at a slow-axis position, then, step to the next slow-axis position), were coherently averaged (Supplementary Fig. 3a, dashed lines). These results suggest that acquisition of multiple B-scans was required to ensure decorrelation between different realizations of noise being averaged. Notably, when nonconsecutive B-scans from the same BM-mode datasets were coherently averaged (i.e., averaging every Δ*T* > 1 B-scans), the noise suppression performance approached the theoretical efficiency with increasing Δ*T* (Supplementary Fig. 3a, light dashed lines).

The distribution of noise power in the spatial-frequency domain provides additional insights into the results in Supplementary Fig. 3a. A circular Gaussian noise is expected to be uniformly distributed across spatial frequencies. This was observed in the single-shot images acquired with both CM- and BM-mode acquisition schemes (Supplementary Fig. 3b, left). The suppressed noise remained uniformly distributed after coherent average of successive OCT volumes (CM-mode), but not successive B-scans (BM-mode) (Supplementary Fig. 3b, right), where noise was suppressed more efficiently at higher spatial frequencies. This result suggests that only the rapidly changing noise at higher spatial frequencies became decorrelated between successive B-scans. In other words, over the shorter time scale associated with frame averaging, there is likely systematic contributions to the system noise (e.g., resulting from fluctuations of laser intensity).


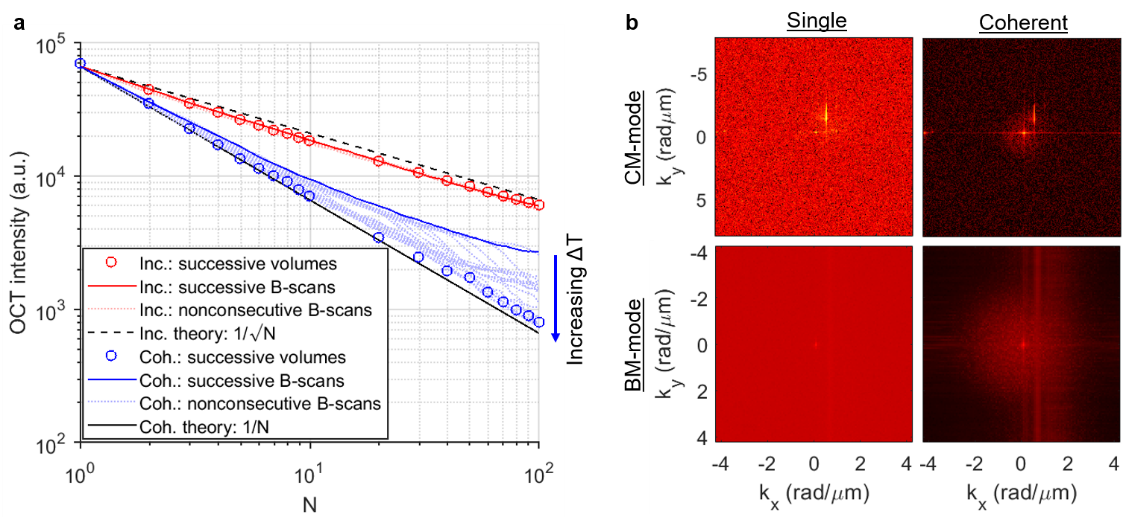


**Supplementary Figure 3 Comparison of averaging schemes for noise suppression. a**, Noise reduction as a function of *N* for successive-volumes (circle), successive-B-scans (solid), and nonconsecutive-B-scans (light dotted) averaging schemes for incoherent (red) and coherent (blue) average. Coherent-average noise reduction with nonconsecutive B-scans approached the optimal efficiency of successive-volumes averaging scheme as Δ*T* increased. **b**, Single-shot and coherent-average noise power in transverse spatial-frequency domain for CM- and BM-mode acquisition schemes. For **a** and **b**, noise data was obtained from an *en face* plane located inside a glass coverslip.

**Supplementary Section IV: RE-OCT reconstruction procedure**

The RE-OCT reconstruction procedure implemented in all experiments is illustrated in a flowchart in Supplementary Fig. 4. First, the space-domain OCT volume from each acquisition was reconstructed by standard procedure (background subtraction, spectrum resampling, dispersion correction, and inverse Fourier transform). Defocus correction was performed on each reconstructed volume via computational image formation procedure (phase registration, bulk demodulation, and computational adaptive optics) as previously described^5^. As a result, each OCT volume has depth-invariant transverse spatial-frequency bandwidth and resolution. For *ex vivo* mouse brain, additional image registration procedure was necessary in order to ensure that individual volumes were phase registered to each other in the presence of sample instability during CM-mode acquisitions (Supplementary Section V).

The phase-registered OCT volumes were coherently averaged to obtain the *coherent-average OCT volume* via:

$\tilde{S}_{\text{avg}}\left(x,y,z\right)=\frac{1}{N}\sum_{i=1}^{N} \tilde{S}_{i}\left(x,y,z\right)$. (S7)

Then, the computational bandwidth expansion (BE) procedure began with computing of the *Fourier-domain OCT volume* via:

$\tilde{S}_{\text{FD,avg}}\left(k_{x},k_{y},z\right)=\text{FT}_{x,y}\left[\tilde{S}_{\text{avg}}\left(x,y,z\right)\right]$, (S8)

where $\text{FT}_{x,y}$ denotes 2D Fourier transform along the $x$ and $y$ dimensions, followed by a magnitude-average across depths about the focal plane $z_{f}$ to obtain the *magnitude spectrum*:

$M\left(k_{x},k_{y}\right)=\frac{1}{21}\sum_{i=-10}^{10} \left|\tilde{S}_{\text{FD,avg}}\left(k_{x},k_{y},z_{f+i}\right)\right|$. (S9)

The depth averaging served to minimize the rapid noisy fluctuations in the magnitude spectrum that are present at a single plane.


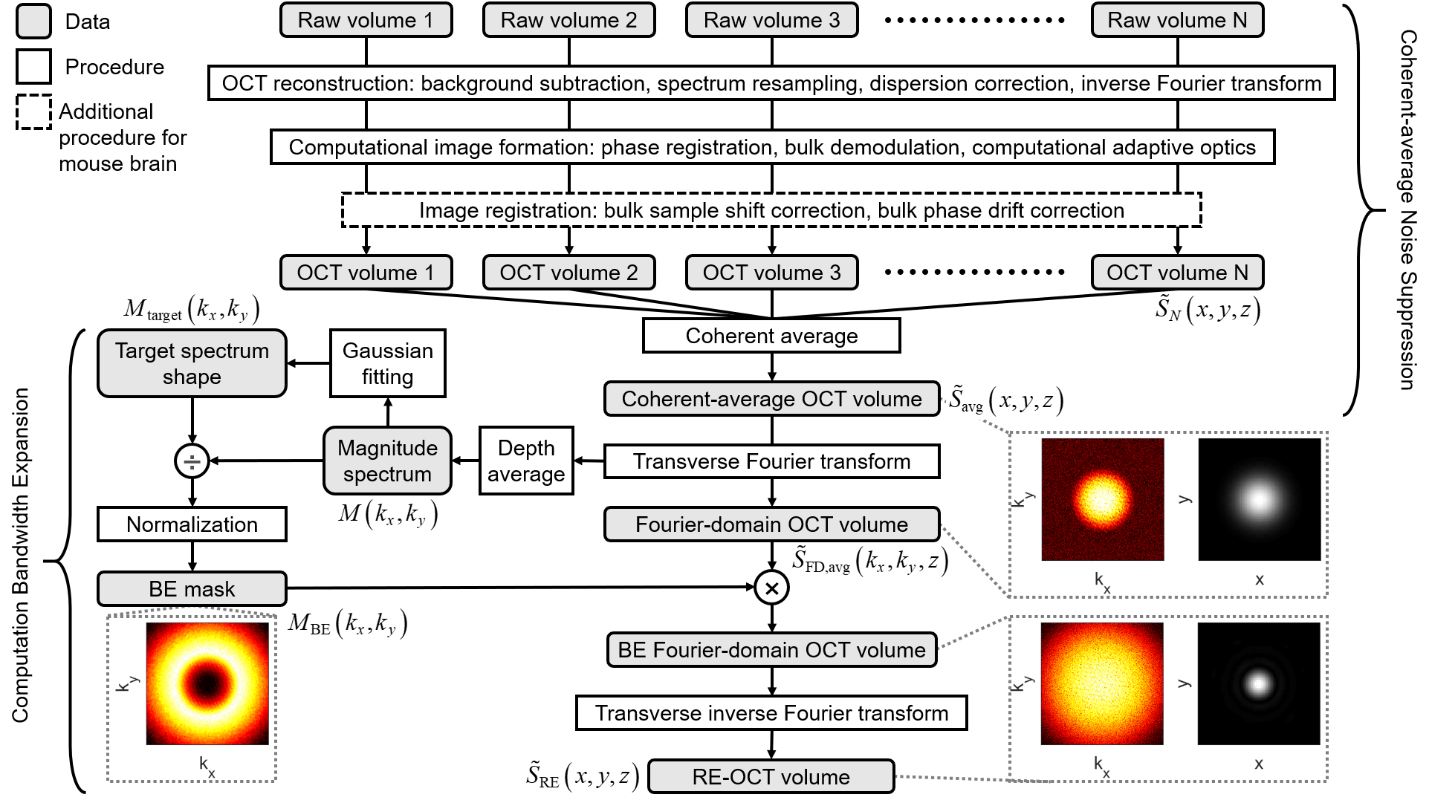


**Supplementary Figure 4 RE-OCT reconstruction procedure.** Individual space-domain OCT volumes were reconstructed and processed for coherent-average noise suppression. Computational BE was performed on the coherent-average OCT volume via magnitude-based deconvolution to obtained the resolution-enhanced RE-OCT volume.

The depth-average *magnitude spectrum* was fit to a Gaussian curve as a function of the radial spatial frequency, $k_{r}=\sqrt{k_{x}^{2}+k_{y}^{2}}$, then, the fit parameters $a$, $c$, and $d$ were used to compute the bandwidth-expanded *target spectrum shape* with a given BE factor, $\varepsilon_{\text{BE}}$, via:

$M_{\text{fit}}\left(k_{r}\right)=a\exp\left(-k_{r}^{2}/c^{2}\right)+d$, (S10)

$M_{\text{target}}\left(k_{x},k_{y}\right)=a\exp\left(-\left(k_{x}^{2}+k_{y}^{2}\right)/\left(\varepsilon_{\text{BE}}c\right)^{2}\right)+d$. (S11)

The *BE mask* was computed from the *target spectrum shape* and the *magnitude spectrum*, then, normalized to ensure that the total signal power would be conserved after computational BE, as follows:

$M_{\text{BE}}\left(k_{x},k_{y}\right)=\frac{1}{\varepsilon_{\text{BE}}^{2}}\frac{M_{\text{target}}\left(k_{x},k_{y}\right)}{M\left(k_{x},k_{y}\right)}$. (S12)

Finally, the resolution-enhanced RE-OCT volume was obtained via magnitude-based deconvolution in the spatial-frequency domain:

$\tilde{S}_{\text{RE}}\left(x,y,z\right)=\text{FT}_{x,y}^{-1}\left[M_{\text{BE}}\left(k_{x},k_{y}\right)\tilde{S}_{\text{FD,avg}}\left(x,y,z\right)\right]$, (S13)

where $\text{FT}_{x,y}^{-1}$ denotes 2D inverse Fourier transform along the $x$ and $y$ dimensions.

**Supplementary Section V: Image registration procedure for *ex vivo* mouse brain**

The *ex vivo* mouse brain in this study experienced both bulk sample shift and bulk phase drift, likely due to the temperature stabilization of the cold mouse brain and the warm mounting agarose (see Methods). In such case, image registration procedure was required to ensure that the scattering signal from different OCT volumes are phase-registered to each other prior to computing the coherent average. First, the bulk sample shift in 3D space was corrected via a Fourier transform-based image translation registration algorithm^6^. Each OCT volume was conjugated to the 1^st^ volume in the spatial-frequency domain, before its bulk spatial shifts relative to the 1^st^ volume along $x$, $y$, and $z$ dimensions were estimated from the peak position of space-domain impulse response via:

$\tilde{S}_{\text{FD},i}\left(k_{x},k_{y},k_{z}\right)=\text{FT}_{x,y,z}\left[\tilde{S}_{i}\left(x,y,z\right)\right]\text{ ; }i=1,2,\ldots,N$, (S14)

$\left(\Delta x_{i},\Delta y_{i},\Delta z_{i}\right)=\mathop{\mathrm{argmax}}\limits_{\left(x,y,z\right)} \left(\text{FT}_{x,y,z}^{-1}\left[\tilde{S}_{\text{FD},i}^{*}\left(k_{x},k_{y},k_{z}\right)\tilde{S}_{\text{FD},1}\left(k_{x},k_{y},k_{z}\right)\right]\right)$, (S15)

where $\text{FT}_{x,y,z}$ and $\text{FT}_{x,y,z}^{-1}$ denote the 3D forward and inverse Fourier transform, and $\tilde{S}_{\text{FD},i}^{*}$ denotes the complex conjugate of $\tilde{S}_{\text{FD},i}$, respectively. Then, the bulk spatial shifts $\Delta x_{i}$, $\Delta y_{i}$, and $\Delta z_{i}$ were applied back to the *i*^th^ volume as phase ramps to spatially register it to the 1^st^ volume via:

$\tilde{S}_{i\text{,shift-corrected}}\left(x,y,z\right)=\text{FT}_{x,y,z}^{-1}\left[\tilde{S}_{\text{FD},i}\left(k_{x},k_{y},k_{z}\right)\exp\left(-j2\pi\left(k_{x}\Delta x_{i}+k_{y}\Delta y_{i}+k_{z}\Delta z_{i}\right)\right)\right]$, (S16)

where $j=\sqrt{-1}$.


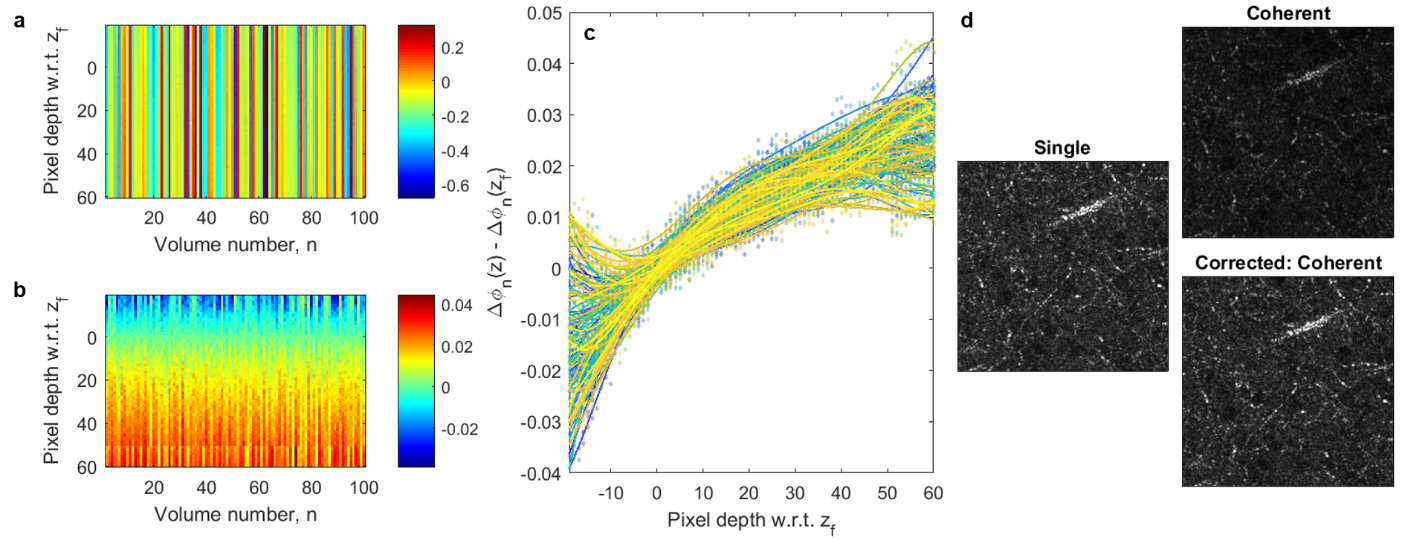


**Supplementary Figure 5 Bulk phase drift correction in *ex vivo* mouse brain. a,** Bulk phase difference between adjacent volumes at each pixel depth. **b**, Bulk phase difference in **a** w.r.t. the focal plane, , to show the depth dependence. **c**, Depth-dependent polynomial curve fits of bulk phase difference for different volumes in **b**. **d**, Single-shot and coherent-average images with and without image registration (same colormap range).

After correcting for the bulk sample shift, a bulk phase difference between adjacent volumes was estimated at each pixel depth $k$ via:

, (S17)

which represents the magnitude-weighted average phase difference across each *en face* plane (Supplementary Fig. 5a). Each of the *i*^th^ phase difference was fit to a 6^th^-order polynomial function as a function of $z$ via linear least-square curve fitting to obtain a depth-dependent phase shift between adjacent volumes (Supplementary Fig. 5c). The cumulative depth-dependent phase drift up to the *i*^th^ volume was removed from each subsequent volume to phase-register it to the 1^st^ volume via:

. (S17)

Supplementary Figure 5d shows the comparison between the coherent-average images of the *ex vivo* mouse brain with and without image registration.

**Supplementary Section VI: Simulation of RE-OCT in silicone phantom**

In order to understand the factors that limit achievable resolution enhancement in RE-OCT, a set of simulated *en face* planes were generated with the same number of pixels (450×450 pixels), transverse FOV (180 µm × 180 µm) and spatial sampling (0.4 µm/pixel) as the silicone phantom datasets in Figs. 1 and 2. The simulated *en face* planes consisted of three components: scattering particles (Supplementary Fig. 6a), silicone background (Supplementary Fig. 6b), and system noise (Supplementary Fig. 6c).


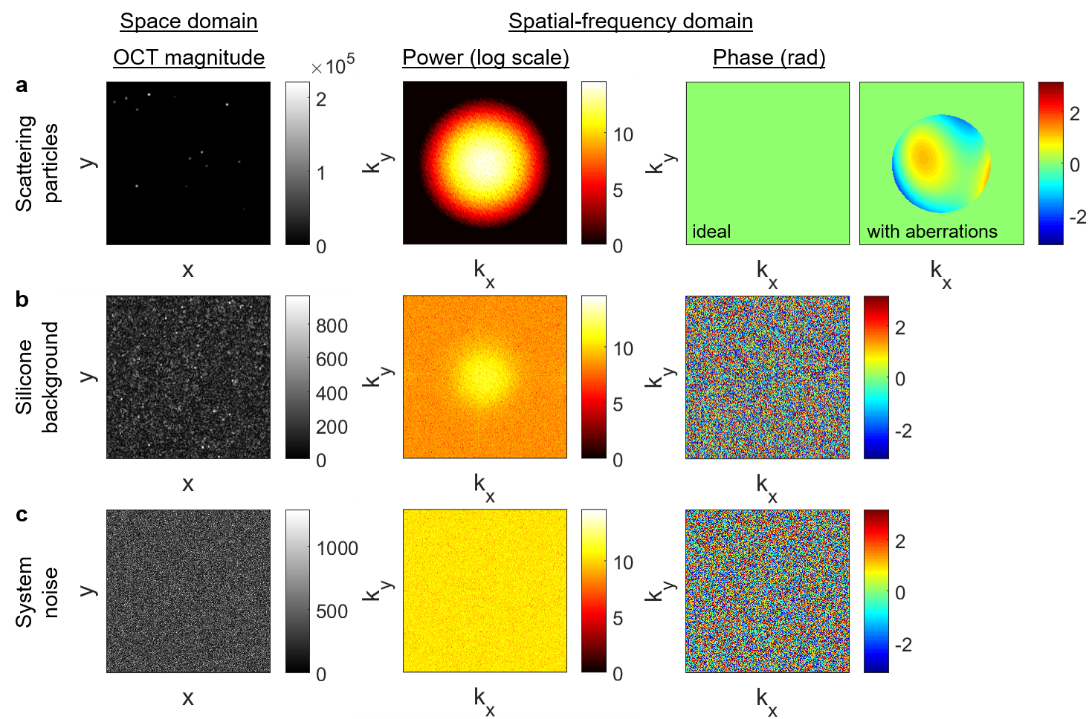


**Supplementary Figure 6 Components of simulated *en face* planes. a**–**c**, Space-domain OCT magnitude image (left), and spatial-frequency-domain power (middle) and phase (right) spectra of simulated scattering particles, silicone background, and system noise, respectively. Phase spectra in **a** show the pupil phase applied to the simulated Gaussian PSFs. Note the power spectrum shape of the silicone background, in contrast to the uniformly distributed spectrum of the random system noise.

Each component was generated as follows:

- **Scattering particles.** 2D symmetric Gaussian point spread functions (PSF) with full width at half-maximum (FWHM) of 2.1 µm, matching the native transverse resolution of the system, and peak magnitudes matching the OCT signal magnitudes of the scattering particles from the focal plane of the silicone phantom datasets (Supplementary Fig. 6a). For the simulated case with optical aberrations, an aberrated phase profile computed with Zernike polynomials were added to the Gaussian PSFs^7^ (Supplementary Fig. 6a, right).
- **Silicone background.** Complex OCT signal of the silicone medium from the focal plane of the silicone phantom datasets. First, the scattering particles were removed from the *en face* image via a magnitude threshold. Then, the gaps left behind at the particle locations were “filled in” by a patch of silicone image from another region (Supplementary Fig. 6b).
- **System noise.** A 450×450 array of circularly symmetric complex random variable with a mean intensity (i.e., magnitude^2^) equivalent to that of the single-shot noise intensity from the focal plane of the silicone phantom datasets in Fig. 1b (Supplementary Fig. 6c). For a coherent-average image across *N* acquisitions, the array of complex random variable was repeatedly generated for *N* iterations and coherently averaged.

A total of six simulated *en face* planes were generated: noise only (scattering particles + system noise), noise with background (scattering particles + silicone background + system noise), and noise-free limit (scattering particles only), each of the three cases with and without optical aberrations. The RE-OCT procedure was performed on each of the simulated *en face* planes as described in Supplementary Section IV. The resolution and SBR results were computed as described in Methods. We note that although physical aperture imposed by the objective lens has not been included in the simulations, the relevant spatial frequencies in our simulations remain within the double-pass pupil of the OCT system used for the presented experimental results (maximum spatial frequency of 6.67 rad/um for our 0.45 NA objective lens).

**Supplementary Section VII: Noise, dynamic range, and phase correlation in the spatial-frequency domain**

System noise limits not only the available DR of the image, but also the spatial-frequency bandwidth over which signal phase remains correlated in spatial frequency. In order to investigate the results observed in Figs. 1c–e over a wider range of noise levels, simulated *en face* planes containing scattering particles and system noise (i.e., noise only case described in Supplementary Section VI) were generated for coherent average over a range of *N* = 1 (Supplementary Fig. 7a) through *N* = 10^5^ (Supplementary Fig. 7b) acquisitions. DR and phase-correlation limit, $k_{\text{phase-corr}}$, in the spatial-frequency domain were computed for each simulated *en face* plane as described in Methods.


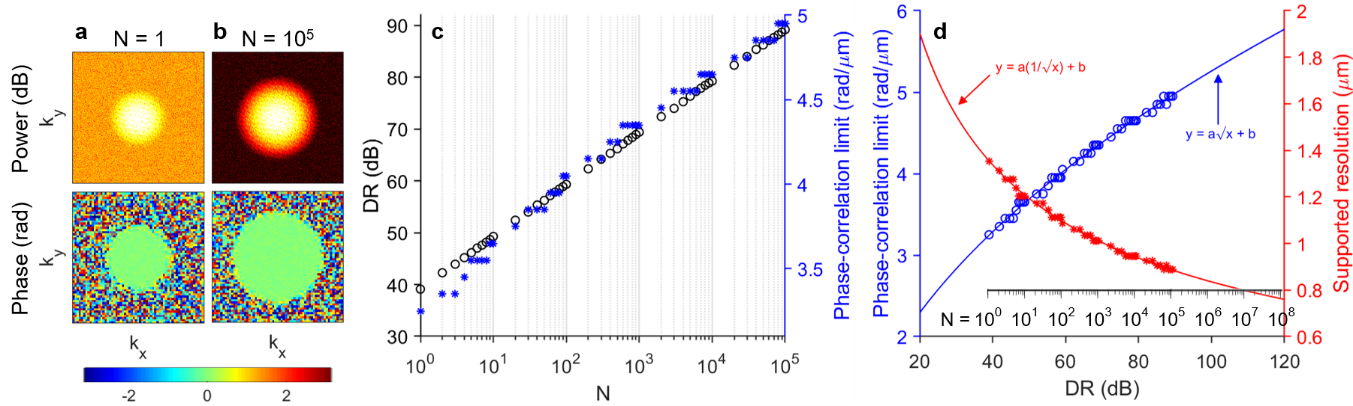


**Supplementary Figure 7 Noise-limited dynamic range and phase-correlation limit. a** and **b**, Power and phase of simulated single-shot (*N* = 1) and coherent-average (*N* = 10^5^) image in transverse spatial-frequency domain. **c**, DR (black) and phase-correlation limit (blue) as a function of *N*. **d**, Phase-correlation limit (blue) and the supported resolution (red) as a function of DR. Both simulation results (marker) and curve-fits (line) are shown. Inset shows corresponding *N* required for the DR values.

Both DR and phase-correlation limit increases as the system noise is further suppressed via coherent average over larger *N* (Supplementary Fig. 7c). For the definition of the phase-correlation limit implemented here (see Methods), the phase-correlation limit corresponds to the spatial frequency at which the signal power is roughly 16 dB above the system noise floor. Given the quadratic drop of the Gaussian spectrum tail on the logarithmic scale, the increase in phase-correlation limit from coherent-average noise suppression becomes less efficient at increasing *N*, even though DR increases linearly with *N* on the logarithmic scale (i.e., noise suppression factor of 1/*N*). This diminishing efficiency can be seen on the plot of phase-correlation limit as a function of DR, where the phase-correlation limit scales with square root of DR (Supplementary Fig. 7d, blue). The phase-correlation limit also provides an estimate of the best possible resolution that the system can support (Supplementary Fig. 7d, red)—given by *λ*/(2NA_max_) and *k*NA_max_ = *k*_phase-corr_, where *k* is the wave number—under the premise that signal from higher spatial frequencies beyond the phase-correlation limit cannot constructively interfere, thus, cannot contribute to the localization of PSF energy in the space domain.

**Supplementary Section VIII: Fundamental limits to resolution enhancement in RE-OCT**

RE-OCT utilizes the framework for resolution enhancement based on gaining extra SNR via coherent-average noise suppression and the *theorem of invariance of information capacity* presented in Supplementary Section I. However, the amount of resolution enhancement in RE-OCT need not be limited to that of the SNR gain obtained through coherent averaging. In fact, the BE factor can be selected to achieve the most optimal combination of DR and resolution, depending on the SNR penalty that can be tolerated in an application. In a general case where any BE factor may be applied, the theoretically achieved RE factor and the accompanying SNR penalty based on Cox and Sheppard’s information capacity framework^1^ is:

$\frac{\text{Res}_{\text{native}}}{\text{Res}_{\text{RE-OCT}}}=\frac{B_{\text{RE}}}{B_{\text{native}}}=\sqrt{\frac{\log_{2}\left(s/n_{\text{avg}}\right)}{\log_{2}\left(s/n_{\text{RE}}\right)}}$, (S19)

where $B_{\text{native}}$ and $B_{\text{RE}}$ denote the isotropic spatial-frequency bandwidth in both $x$ and $y$ dimensions before and after computational BE, respectively. In principle, the resolution is enhanced by as much as the bandwidth is expanded (i.e., RE factor = BE factor), and the bandwidth can be expanded by as much as one is willing to sacrifice the SNR (according to the *theorem of invariance of information capacity*). The SNR penalty (in dB) in exchange for a given BE factor is $10\log_{10}\left( n_{\text{avg}}/n_{\text{RE}}\right)$, where $n_{\text{RE}}\geq n_{\text{avg}}$ for BE factor ≥ 1. This is the minimum SNR that must be ‘earned’ via coherent average if one were to maintain the original single-shot SNR in the final RE-OCT image.


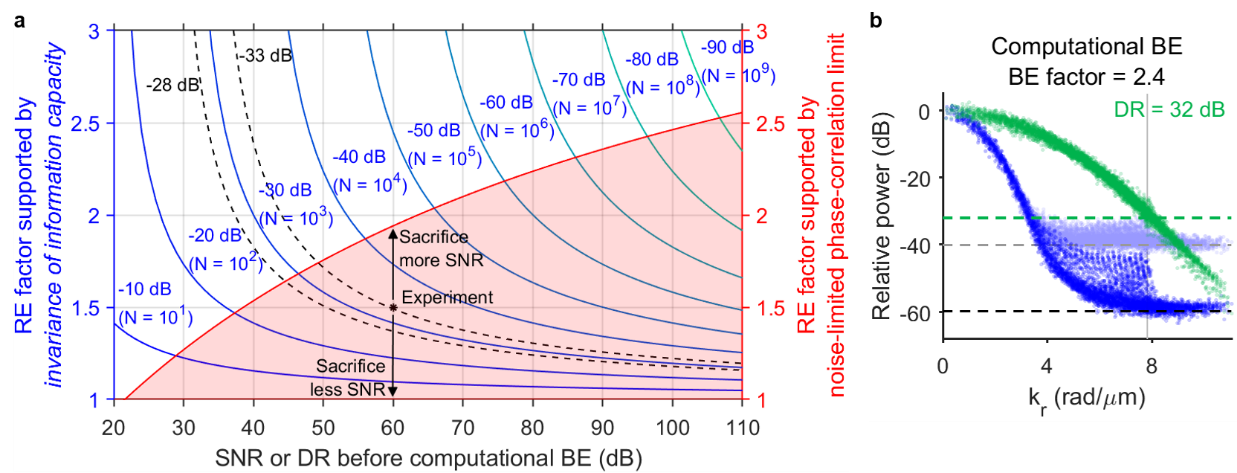


**Supplementary Figure 8 Fundamental limits to resolution enhancement in RE-OCT.** **a**, RE factor as a function of initial SNR (or DR in the spatial-frequency domain) before computational BE supported by the *theorem of invariance of information capacity* at different amount of SNR penalty (blue) and the additional practical limit imposed by the noise-limited phase-correlation limit (red). Each blue curve is labeled with the SNR penalty and the number of acquisitions *N* required in coherent-average noise suppression to fully compensate for the loss. Shaded region under the red curve represents the practical “RE-OCT operating range”. Black marker indicates the experimental performance in silicone phantom in Figs. 1 and 2, where in principle, the performance may be adjusted up or down within the operating range to prioritize resolution or SNR, respectively. **b**, Relative power as a function of radial spatial frequency of the single-shot (light blue, DR = 40 dB), coherent-average (dark blue, DR = 60 dB), and RE-OCT (green, DR = 32 dB) images of the silicone phantom (Fig. 2a). An estimate of -28 dB in SNR has been sacrificed during the computational BE procedure to produce the RE-OCT image in Fig. 2.

The resolution enhancement supported by Cox and Sheppard’s information capacity framework^1^ with different amounts of SNR penalty is shown as a function of the initial SNR before computational BE (Supplementary Fig. 8a, blue). Ironically, starting with a lower initial SNR supports larger RE factor for a given dB sacrificed (i.e., each individual curve has a decreasing trend). However, starting with a higher SNR means there is more SNR to sacrifice by computational BE before the final SNR of the bandwidth-expanded image drops below 0 dB. More importantly, this fundamental limit based on the *theorem of invariance of information capacity* does not account for the implication of noise on the phase correlation in the spatial-frequency domain—that is, the impact of phase decorrelation on disrupting the constructive interference that is required to produce the optimal resolution in space. The SNR-limited phase-correlation limit, *k*_phase‑corr_, in the spatial-frequency domain imposes another upper limit to the best possible resolution that the system can support, based on the spatial-frequency bandwidth over which phase remains correlated (Supplementary Fig. 7d in Supplementary Section VII). Taking the simulation results in Supplementary Fig. 7d and Res_native_ = 2.1 µm, the resolution enhancement supported by the noise-limited phase-correlation limit is shown (in red) on top of the theoretical information-capacity limit in Supplementary Fig. 8a. Note that the simulation results in Supplementary Fig. 7d only account for the effects of system noise; there are other factors that can disrupt phase correlation in the spatial-frequency domain, thus, further limiting the achievable resolution enhancement, in practice (as discussed in the main manuscript).

The red shaded region under the curve represents the practical “RE-OCT operating range”. The experimental performance in silicone phantom (i.e., a coherent-average DR of 60 dB going into computational BE with a BE factor of 2.4, resulting in an RE factor of 1.5) is indicated by a black marker. In principle, this asterisk can be flexibly moved up (to achieve better resolution improvement while sacrificing more SNR) or down (to preserve more SNR while achieving less resolution improvement) within the shaded region, at a given initial coherent-average SNR going into computational BE (Supplementary Fig. 8a, black arrows). The experimental performance suggests that roughly 33 dB in SNR was sacrificed during the computational BE procedure, where the -33 dB dashed curve intersects the black marker. This SNR penalty is slightly more than the -28 dB estimate based on the relative power spectrum of the bandwidth-expanded versus coherent-average image (Supplementary Fig. 8b). These results suggest that there may be slight discrepancies between the true SNR in the space domain of the RE-OCT image (which we do not have an experimental measurement of) and the estimated DR in the spatial-frequency domain.

**Supplementary Movie 1 Caption**

**Resolution-enhanced (RE)-OCT in silicone phantom with increasing bandwidth expansion (BE) factor.** **a**, Single-shot, coherent-average, BE single-shot, and RE-OCT power spectrums (log scale) and space-domain *en face* OCT image with zoomed PSF (linear scale). Resolution and SBR represent mean ± standard deviation of measurements from 11 particles. Scale bars, 40 µm (*en face* image) and 2 µm (zoomed PSF). **b** and **c**, Cross-sectional profiles of zoomed PSF in a on peak-normalized linear and log scales.

**Supplementary Movie 2 Caption**

**Resolution-enhanced (RE)-OCT in fibrous collagen gel with increasing bandwidth expansion (BE) factor. a**, Single-shot and RE-OCT *en face* OCT images with zoomed insets regions indicated by boxes. Scale bars, 40 µm (full) and 20 µm (zoomed). **b** and **c**, Cross-sectional profiles of a line connecting from small to larger green dots in the green zoomed insets in a on linear and peak-normalized log scales.

**Supplementary Movie 3 Caption**

**Resolution-enhanced (RE)-OCT in the cortex of *ex vivo* mouse brain with increasing bandwidth expansion (BE) factor.** **a**, Single-shot and RE-OCT *en face* OCT images with zoomed insets regions indicated by boxes. Images were taken in the first cortical layer at approximately 100 µm below surface. × markers in the green zoomed insets indicate one of the neurons, which appear as darker circles due to weak OCT scattering. Green inset shows that neuron in BE single-shot image was barely discernible due to the SNR penalty without coherent-average noise suppression. Scale bars, 40 µm (full) and 20 µm (zoomed). **b** and **c**, Cross-sectional profiles of a line connecting from small to large green dots in the green zoomed insets in a on linear and peak-normalized log scales. The green × marker indicates its corresponding position on the image.

**References**

1 Cox, I. J. & Sheppard, C. J. R. Information capacity and resolution in an optical system. *J. Opt. Soc. Am. A* **3**, 1152-1158 (1986).

2 Sheppard, C. J. R. & Larkin, K. G. Information capacity and resolution in three-dimensional imaging. *Optik* **113**, 548-550 (2003).

3 Baumann, B. *et al.* Signal averaging improves signal-to-noise in OCT images: But which approach works best, and when? *Biomed. Opt. Express* **10**, 5755-5775 (2019).

4 Pfeiffer, T. *et al.* Flexible A-scan rate MHz-OCT: efficient computational downscaling by coherent averaging. *Biomed. Opt. Express* **11**, 6799-6811 (2020).

5 Mulligan, J. A., Feng, X. & Adie, S. G. Quantitative reconstruction of time-varying 3D cell forces with traction force optical coherence microscopy. *Sci. Rep.* **9**, 4086 (2019).

6 S., R. B. & Chatterji, B. N. An FFT-Based Technique for Translation, Rotation, and Scale-Invariant Image Registration. *IEEE Trans. Image Process.* **5**, 1266-1271 (1996).

7 Adie, S. G., Graf, B. W., Ahmad, A., Carney, P. S. & Boppart, S. A. Computational adaptive optics for broadband optical interferometric tomography of biological tissue. *Proc. Natl. Acad. Sci. U.S.A.* **109**, 7175-7180 (2012).
